# Antiviral Susceptibility of Swine-Origin Influenza A Viruses Isolated from Humans, United States

**DOI:** 10.3201/eid3011.240892

**Published:** 2024-11

**Authors:** Rongyuan Gao, Philippe Noriel Q. Pascua, Anton Chesnokov, Ha T. Nguyen, Timothy M. Uyeki, Vasiliy P. Mishin, Natosha Zanders, Dan Cui, Yunho Jang, Joyce Jones, Juan De La Cruz, Han Di, Charles Todd Davis, Larisa V. Gubareva

**Affiliations:** Centers for Disease Control and Prevention, Atlanta, Georgia, USA

**Keywords:** influenza, viruses, swine-origin influenza A virus, human infections, antivirals, baloxavir marboxil, antimicrobial resistance, pandemic risk assessment, monoclonal antibodies, hemagglutinin, IRINA, United States

## Abstract

Since 2013, a total of 167 human infections with swine-origin (variant) influenza A viruses of A(H1N1)v, A(H1N2)v, and A(H3N2)v subtypes have been reported in the United States. Analysis of 147 genome sequences revealed that nearly all had S31N substitution, an M2 channel blocker-resistance marker, whereas neuraminidase inhibitor–resistance markers were not found. Two viruses had a polymerase acidic substitution (I38M or E199G) associated with decreased susceptibility to baloxavir, an inhibitor of viral cap-dependent endonuclease (CEN). Using phenotypic assays, we established subtype-specific susceptibility baselines for neuraminidase and CEN inhibitors. When compared with either baseline or CEN-sequence–matched controls, only the I38M substitution decreased baloxavir susceptibility, by 27-fold. Human monoclonal antibodies FI6v3 and CR9114 targeting the hemagglutinin’s stem showed variable (0.03 to >10 µg/mL) neutralizing activity toward variant viruses, even within the same clade. Methodology and interpretation of laboratory data described in this study provide information for risk assessment and decision-making on therapeutic control measures.

Influenza A viruses are enzootic in swine populations worldwide. All swine influenza A viruses are 1 of 3 antigenic subtypes—H1N1, H1N2, and H3N2—and distinct differences in evolutionary patterns exist between North American and Eurasian viruses (*1*). Because pigs are susceptible to influenza viruses circulating in humans, birds, and other species, they are considered mixing vessels (*2*). Accordingly, co-infection with viruses from different species can generate reassortants with zoonotic and pandemic potential (*2*–*4*). Beginning in the late 1990s, swine influenza viruses containing gene segments from human, avian, and classical swine viruses became widespread in pigs in North America (*4*,*5*). Those viruses contained the triple-reassortment internal gene (TRIG) cassette consisting of human-lineage polymerase basic 1 (PB1); avian-lineage polymerase basic 2 (PB2) and polymerase acidic (PA); and classical swine-lineage nucleoprotein (NP), matrix (M), and nonstructural (NS) segments (*1*). This TRIG cassette pairs with various hemagglutinin (HA) and neuraminidase (NA) gene combinations derived from classical swine or human lineages (*1*,*4*,*5*).

Under World Health Organization (WHO) International Health Regulations (2005), human infections by swine-origin influenza A viruses are reportable (*6*). In 2007, the US Centers for Disease Control and Prevention (CDC) began a similar national notification mandate (*7*). Previously, such cases had been rare in the United States (*8*). However, a novel swine-origin influenza A(H1N1) virus emerged in 2009 in North America, causing the first influenza pandemic of the 21st Century. Genetically, that virus, designated A(H1N1)pdm09, closely resembled North American triple-reassortant swine A(H1N1) viruses but with NA and M segments from Eurasian swine lineage (*9*,*10*). By 2010, A(H1N1)pdm09 had replaced seasonal influenza A(H1N1) viruses previously circulating in humans. Furthermore, humans have repeatedly reintroduced A(H1N1)pdm09 into pigs (*11*,*12*). Those reverse zoonoses, followed by reassortment in pigs, increased the diversity of swine viruses, creating new genotypes of unknown epidemiologic implications. Swine-origin viruses that cause human infections are called variant viruses to distinguish them from seasonal viruses and are denoted as A(HxNx)v (*13*).

In 2012, influenza A(H3N2)v viruses containing A(H1N1)pdm09-derived M gene (M-pdm09) caused a large multistate outbreak of >300 human infections in the United States; M-pdm09 is now dominant in swine influenza A viruses of all subtypes (*14*,*15*). Moreover, the A(H1N1)pdm09-derived N1 segment (N1-pdm09) has become 1 of 4 cocirculating NA lineages, alongside classical swine-lineage N1 (N1-classical) and 2 human N2 lineages, N2-1998 and N2-2002 (*16*). N2-1998 and N2-2002 resulted from introductions of human influenza A(H3N2) viruses into pigs in 1998 and 2002 (*4*,*17*). Other gene segments from the A(H1N1)pdm09 lineage and some from live-attenuated influenza vaccine (LAIV) strains used in pigs during 2017–2020 have been detected sporadically (*16*,*18*,*19*). Variant virus infections in the United States are mostly associated with attendance at agricultural fairs or swine exhibitions (*20*). However, concerns remain that these viruses could cause broader spillover events or even trigger a new pandemic.

CDC and other laboratories of the WHO Global Influenza Surveillance and Research System regularly conduct virologic characterization of emerging zoonotic viruses. Laboratory data are used for risk assessment, pandemic preparedness, and antiviral treatment recommendations (*21*,*22*). Three classes of influenza antiviral drugs are approved by the Food and Drug Administration to manage influenza A virus infections: M2 channel blockers, NA inhibitors (NAIs), and a PA cap-dependent endonuclease (CEN) inhibitor (PA-CENI) (*23*). However, genetic changes caused by spontaneous mutations, gene reassortment, or selective pressure (caused by antiviral treatment) might compromise the usefulness of those drugs. For example, CDC recommends against the use of M2 blockers for seasonal influenza A viruses because of resistance, a characteristic independently acquired by A(H1N1)pdm09 and A(H3N2) subtypes. All variant viruses containing M-pdm09 are resistant to M2 blockers because of the presence of an S31N substitution in the M2 protein. In the United States, 3 NAIs (oseltamivir, zanamivir, and peramivir) are approved, and laninamivir is additionally approved in Japan only. The emergence and global spread of oseltamivir-resistant seasonal H1N1 virus in 2008–2010 and reports of community spread of oseltamivir-resistant A(H1N1)pdm09 viruses (*24*,*25*) demonstrate that broad circulation of antiviral resistant influenza virus is an ongoing public health concern.

The continuing pandemic threat posed by animal-origin influenza A viruses necessitates closely monitoring their antiviral susceptibilities, but phenotypic data for variant viruses are limited, and their interpretation is ill-defined. Here, we describe the testing algorithm and interpretation of phenotypic data for variant viruses collected in the United States since 2013.

## Methods

### Reagents

We dissolved NAIs (Biosynth, https://www.biosynth.com) oseltamivir carboxylate (oseltamivir), zanamivir, peramivir, and laninamivir in sterile distilled water. We dissolved baloxavir acid (MedChem Express, https://www.medchemexpress.com), an active metabolite of prodrug baloxavir marboxil, in dimethyl sulfoxide.

We purchased the broadly cross-reactive HA-stem targeting monoclonal antibodies (mAbs) FI6v3 and CR9114 from Creative Biolabs, Inc. (https://www.creativebiolabs.net). We collected antiserum from ferrets at 28 days postinfection and treated it with receptor-destroying enzyme (Denka Seiken, https://www.denka.co.jp) before use.

### Viruses

Variant influenza viruses were submitted by US public health laboratories to the WHO Collaborating Center for Surveillance, Epidemiology and Control of Influenza at CDC and propagated in MDCK cells (ATCC, https://www.atcc.org) according to standard procedures (*26*). We used the CDC antiviral susceptibility reference virus panels (International Reagent Resource FR-1755 and FR-1678) as controls in phenotypic assays. Handling and testing of variant viruses were conducted in Biosafety Level 2 enhanced laboratories.

### Next-Generation Sequencing and Analysis

We obtained whole-genome sequences by using the Illumina next-generation sequencing platform, analyzed by the iterative refinement meta-assembler (*27*), and deposited into GISAID (https://www.gisaid.org) (Appendix). We aligned sequences by using MAFFT version 7 (*28*).

### Antiviral Susceptibility Assays

We examined NAI susceptibility using the fluorescent NA-Fluor kit (Applied Biosystems) (*29*). We assessed baloxavir susceptibility using the influenza replication inhibition neuraminidase-based assay (IRINA) in MDCK-SIAT1 cells (*30*). We determined the 50% inhibitory concentration (IC_50_) and 50% effective concentration (EC_50_) by curve-fitting analysis using nonlinear regression (*29*,*30*).

### Virus Neutralization and HA Antigenic Analysis

To assess the neutralization activity of mAbs, we preincubated 2-fold serially diluted mAb (starting at 10 µg/ml) with virus for 1 hour. We performed IRINA as was done for baloxavir susceptibility testing. We assessed HA antigenicity by hemagglutination inhibition (HI) using 0.5% turkey red blood cells (Lampire Biological Laboratories, https://www.lampire.com) according to standard procedures (*26*) and subsequently by IRINA as previously described (*30*).

## Results

### Genome Sequence Analysis for Molecular Markers of Decreased Drug Susceptibility

During January 2013–April 2024, a total of 167 human infections caused by variant viruses were reported in 22 states across the United States; 17 cases were A(H1N1)v, 34 were (H1N2)v, and 116 were A(H3N2)v (*20*). In addition, 4 A(H1N1)pdm09 viruses from 2021 were recently reclassified as variant viruses based on their HA (*12*). We interrogated the deduced M2, NA, and PA protein sequences of 147 variant viruses to identify amino acid substitutions (molecular markers) associated with antiviral resistance or reduced antiviral susceptibility (Appendix Table 1).

Molecular markers of M2 blocker resistance are well defined; thus, sequence analysis is the primary method to determine susceptibility to this class of influenza antivirals (*31*). All but 1 variant virus had an M-pdm09 segment encoding a resistance-conferring S31N (Appendix Table 1). The exception—A/Hawaii/28/2020 (H3N2)v—had an M segment from the A(H3N2) component of a swine LAIV (*19*) and lacked M2 resistance markers.

Next, we examined sequence data for markers of NAI and PA-CENI resistance that are subtype-specific (*32*,*33*). None of the variant viruses had known markers of resistance to any NAI. However, A/Iowa/02/2021 (H1N1)v had S247N, a substitution that reduces oseltamivir inhibition for A(H5N1) (*34*) but not A(H1N1)pdm09 viruses (*35*) (Appendix Table 1). When analyzing the PA-CEN domain (Appendix Table 1), we identified 2 substitutions associated with decreased baloxavir susceptibility (*33*). I38M, together with the wild-type sequence, was present as a mixed virus population in A/Iowa/33/2017 (H1N1)v (Appendix Table 1) (*36*). The other substitution, E199G, was found in A/New Jersey/53/2015 (H3N2)v. In addition, all 3 subtypes had either serine (S) or proline (P) at position 28. In a seasonal A(H3N2) virus, L28P conferred 2.5-fold reduced baloxavir susceptibility (*37*). Finally, A/Iowa/04/2013 (H3N2)v had a rare I120V substitution, the effect of which was unknown; however, I120T reportedly decreased susceptibility to the PA-CENI, L-742,001 (*38*).

### Assessment of Virus Susceptibility to NAIs

We assessed virus susceptibilities to NAIs using the surrogate phenotypic assay, NA inhibition. Conventionally, influenza A viruses are classified as displaying normal, reduced, or highly reduced inhibition if their IC_50_ is increased by <10-fold (normal), 10- to 100-fold (reduced), or >100-fold (highly reduced) over the subtype-specific median IC_50_ (baseline) (*32*). Therefore, our first task was to establish the variant viruses’ baseline susceptibility. We assembled a panel of 53 viruses available for phenotypic testing that represented the 3 antigenic subtypes collected during 2007–2024 to test their susceptibility to each NAI (Appendix Table 2). We included the A/California/07/2009 (H1N1)pdm09 virus (CA/09) to represent the swine-origin virus that caused the 2009 pandemic.

The enzyme activity of all viruses was potently inhibited by oseltamivir, zanamivir, peramivir, and laninamivir; most IC_50_ fell in a sub-nanomolar range (Appendix Table 2). Among the 3 subtypes, A(H1N2)v exhibited 2- to 4-fold higher median IC_50_, particularly for zanamivir and laninamivir, whereas A(H1N1)v produced 2- to 4-fold higher oseltamivir IC_50_ (Figure, panel A; Appendix Table 2). This analysis exemplifies the utility of subtype-specific baseline values for data interpretation, which we then subsequently used to conduct within-subtype analyses and determine whether individual viruses exhibited reduced antiviral inhibition. A/Iowa/02/2021 (H1N1)v (which had S247N) exhibited 2- to 3-fold increased IC_50_, which is interpreted as normal inhibition (Table 1; Appendix Table 2). A similar outcome was attained when it was compared to its closest NA sequence–matched control or CA/09. Of note, an I427V substitution carried by a 2012 A(H1N1)v virus (A/Missouri/12/2012) conferred 17- to 21-fold reduced inhibition by oseltamivir when compared with the subtype-specific median and CA/09 IC_50_s. To further demonstrate the value of subtype-specific baseline, we next analyzed results for an A(H3N2)v virus with S247P, A/Ohio/88/2012 (Table 1; Appendix Table 2) (*15*). That virus displayed reduced inhibition by oseltamivir and zanamivir using either baseline or a sequence-matched control as comparator.

**Figure:**
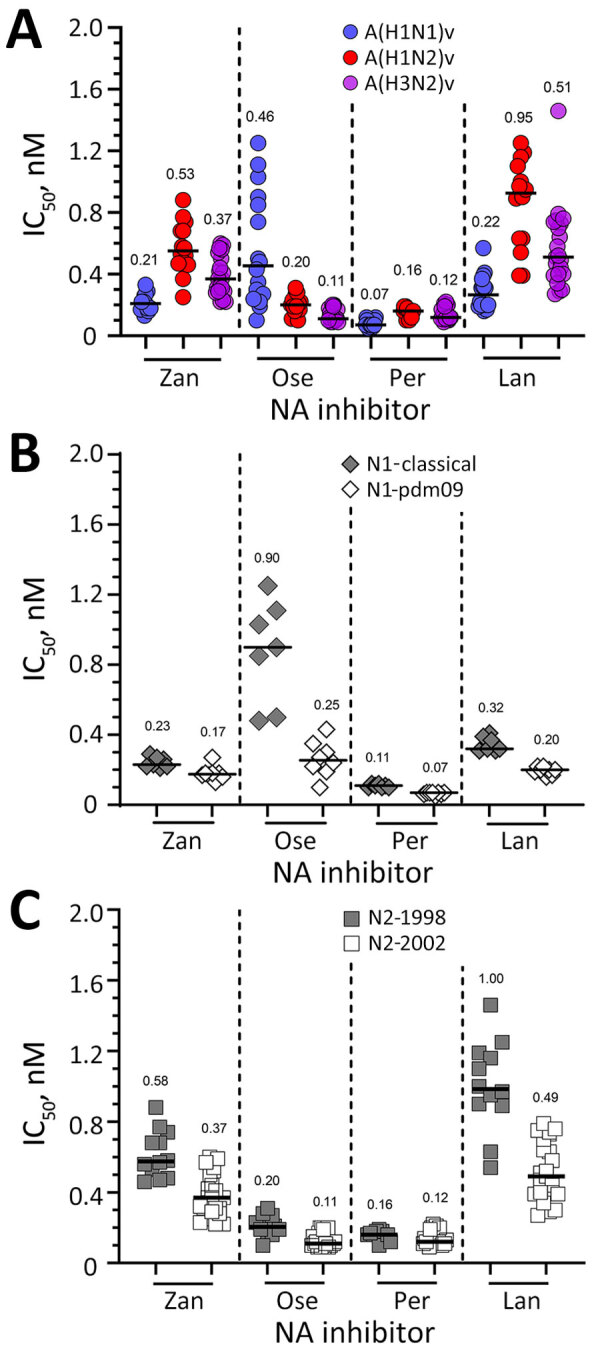
Susceptibility of variant viruses to NA inhibitors based on subtype and NA lineage in study of antiviral susceptibility of swine-origin influenza A viruses isolated from humans, United States. A) Susceptibility of A(H1N1)v (n = 15), A(H1N2)v (n = 14), and A(H3N2)v (n = 21) viruses to NA inhibitors determined in a fluorescence-based assay (*29*). The IC_50_s of viruses lacking known or suspected molecular markers that reduce inhibition by NA inhibitors were used to calculate the subtype-specific median IC_50_s (baseline susceptibility). B, C) Effect of NA lineage on inhibition by NA inhibitors. IC_50_s obtained in NA inhibition assay were grouped according to their respective NA lineage: N1-classical (n = 7, closed diamond), N1-pdm09 (n = 8, open diamond), N2-1998 (n = 12, closed square), or N2-2002 (n = 23, open square). Horizontal bars and numbers indicate median IC_50_s. IC_50_, 50% inhibitory concentration; Lan, laninamivir; NA, neuraminidase; Ose, oseltamivir; Per, peramivir; Zan, zanamivir.

**Table 1:** NA inhibitor susceptibility of variant influenza viruses in a fluorescence-based neuraminidase inhibition assay in study of antiviral susceptibility of swine-origin influenza A viruses isolated from humans, United States*

Influenza A virus	Amino acid change in neuraminidase†	Mean IC_50_ + SD, nM (fold change)			
		Zanamivir	Oseltamivir	Peramivir	Laninamivir
A(H1N1)v median IC_50_, n = 15		0.21	0.43	0.07	0.22
A/Iowa/23/2020	–	0.18 + 0.03 (1; 1)	0.27 + 0.01 (1; 1)	0.07 + 0.01 (1; 1)	0.22 + 0.02 (1; 1)
A/Iowa/02/2021‡	S247N	0.33 + 0.02 (2; 2)	1.05 + 0.18 (2; 4)	0.20 + 0.01 (3; 3)	0.57 + 0.03 (3; 3)
A/Missouri/12/2012§	I427V	0.24 + 0.06 (1; NA)	7.50 + 0.73 (17; NA)¶	0.07 + 0.01 (1; NA)	0.14 + 0.02 (1; NA)
A(H1N2)v median IC_50_, n = 14		0.55	0.20	0.16	0.92
A(H3N2) Median IC_50_, n = 21		0.37	0.11	0.12	0.51
A/Ohio/83/2012	–	0.42 + 0.12 (1; 1)	0.11 + 0.02 (1; 1)	0.13 + 0.02 (1; 1)	0.51 + 0.05 (1; 1)
A/Ohio/88/2012#	S247P	34.78 + 5.40 (94; 83)	5.09 + 1.01 (46; 46)	0.20 + 0.02 (2; 2)	4.45 + 0.52 (9; 9)
Reference seasonal viruses**					
A/Illinois/45/2019 (H1N1)pdm09	–	0.15 + 0.03	0.19 + 0.03	0.05 + 0.01	0.16 + 0.03
A/Pennsylvania/46/2015 (H3N2)	–	0.24 + 0.04	0.15 + 0.03	0.09 + 0.02	0.36 + 0.06

*Each virus was tested in >3 independent experiments to determine IC_50_ value. Fold change values are given for the IC_50_ of variant virus containing amino acid substitution relative to subtype-specific median IC_50_ and wild-type NA sequence-matched virus IC_50_, respectively. Viruses lacking known neuraminidase markers were used to determine the median IC_50_ (Appendix Table 2). Dashes indicate the absence of known or suspected molecular markers that reduce susceptibility to neuraminidase inhibitors. IC_50_, 50% inhibitory concentration; NA, not applicable. †The head domain of the neuraminidase that encompasses amino acid residues 82 to 470. Straight numbering is used to show amino acid substitutions. Residue 247 in N1 corresponds to residue 246 in N2. ‡A/Iowa/02/2021 (H1N1)v also contains N188S and V398I neuraminidase substitutions not present in the wild-type control virus, A/Iowa/23/2020 (H1N1)v. §Wild-type control virus for A/Missouri/12/2012 (H1N1)v was not available for comparison. ¶The I427V neuraminidase substitution confers reduced inhibition by oseltamivir (17-fold increase) when compared with the A(H1N1)v median IC_50_ but normal inhibition (8-fold increase) when compared with the N1-classical IC_50_. #This virus was tested previously (*15*). **Source: Centers for Disease Control and Prevention Neuraminidase Inhibitor Susceptibility Reference Virus Panel (IRR: FR-1755 ver3) (https://www.isirv.org/site/index.php/reference-panel).

To examine whether baseline IC_50_ values are NA-lineage specific, we grouped viruses according to their NA clade: N1-classical (n = 7), N1-pdm09 (n = 8), N2-1998 (n = 12), and N2-2002 (n = 23). Viruses possessing N1-classical had ≈4-fold higher median IC_50_ for oseltamivir compared with those containing N1-pdm09 (Figure, panel B; Appendix Table 2). Viruses possessing N2-1998 had ≈2-fold higher median IC_50_ for oseltamivir and laninamivir compared with those with N2-2002 (Figure, panel C). Overall, the observed interlineage differences were small but might affect interpretation of testing outcomes, particularly for N1 subtype against oseltamivir. For example, A/Missouri/12/2012 (H1N1)v would be reported either as displaying normal or reduced inhibition by oseltamivir, if the N1-classical baseline (8-fold) or the A(H1N1)v subtype-specific baseline (17-fold) was used for comparison (Table 1; Figure, panel B; Appendix Table 2).

### Assessment of Susceptibility to Baloxavir

Susceptibility to baloxavir is assessed using cell culture-based assay. To report decreased susceptibility, the provisional cutoff was set at >3-fold above the subtype-specific baseline (*39*–*41*). Therefore, we first established baseline susceptibility using the same virus panel used previously except for 2 viruses with PA substitutions I38M and E199G. We tested viruses in the single-cycle replication assay IRINA (*30*) where the median EC_50_ values were 0.75 nM for A(H1N1)v, 0.98 nM for A(H1N2)v, and 1.27 nM for A(H3N2)v subtypes (Appendix Table 3). Of note, most A(H1N1)v viruses (11 of 16) had PA-pdm09, whereas PA-TRIG was dominant among A(H1N2)v and A(H3N2)v viruses. This finding raised the question of whether PA-pdm09 contributes to a somewhat lower A(H1N1)v baseline. However, we observed no apparent differences between A(H1N1)v viruses with PA-pdm09 or PA-TRIG (EC_50_ ranges 0.44–1.36 nM for PA-pdm09 and 0.50–1.58 nM for PA-TRIG) (Appendix Table 3). Therefore, the PA-lineage does not affect the susceptibility of US variant viruses to baloxavir. Of note, 2 viruses, A/Michigan/288/2019 (H1N1)v and A/Hawaii/28/2020 (H3N2)v, contained the LAIV virus–derived PA segment (*19*). Of those, 1 virus, A/Michigan/288/2019 (H1N1)v, exceeded the 3-fold provisional threshold compared to its subtype-specific baseline; however, substitutions in its PA-CEN domain associated with reduced baloxavir susceptibility were not present (Appendix Table 3).

A total of 11 viruses (e.g., A/Texas/14/2008) from all 3 subtypes shared the PA-CEN domain with identical amino acid sequence (Appendix Table 3). For those viruses, we again observed that A(H1N1)v viruses produced somewhat lower EC_50_ compared with A(H3N2)v (i.e., 0.54–0.93 nM vs. 1.0–1.85 nM). Therefore, A(H1N1)v viruses in this panel, except A/Michigan/288/2019, seemed slightly more susceptible to baloxavir. This difference is potentially because of differences in early-stage replication kinetics. Within each subtype, we noted no apparent difference in baloxavir susceptibility between viruses having S or P at residue 28 (Appendix Table 3).

A/Iowa/33/2017 (H1N1)v with I38M showed 27-fold decreased baloxavir susceptibility compared with the subtype-specific baseline and 15-fold decreased baloxavir susceptibility compared with the sequence-matched control (Table 2; Appendix Table 3). The observed effect is similar to that of I38M in a seasonal influenza A(H3N2) virus. Conversely, E199G found in A/New Jersey/53/2015 had no effect on baloxavir susceptibility when compared with either the subtype-specific baseline or a sequence-matched control (Table 2). I120V also did not alter baloxavir susceptibility of A/Iowa/04/2013 (Appendix Table 3).

**Table 2:** Baloxavir susceptibility of variant influenza viruses in cell culture-based IRINA in study of antiviral susceptibility of swine-origin influenza A viruses isolated from humans, United States*

Influenza viruses	Amino acid change in PA-CEN†	Mean EC_50_ + SD, nM (fold change)
A(H1N1v) median EC_50_, n = 15		0.75
A/Iowa/33/2017 clone 1‡	–	1.33 + 0.07 (2; 1)
A/Iowa/33/2017 clone 2‡	I38M	19.98 + 2.17 (27; 15)
A(H1N2v) median EC_50_, n = 14	–	0.98
A(H3N2v) median EC_50_, n = 21		1.27
A/Michigan/84/2016	–	1.53 + 0.31 (1; 1)
A/New Jersey/53/2015	E199G	1.50 + 0.17 (1; 1)
Reference seasonal viruses¶		
A/Louisiana/50/2017(H3N2)	–	1.08 + 0.17 (NA; 1)
A/Louisiana/49/2017(H3N2)	I38M	14.06 + 2.96 (13)

*Each virus was tested in >3 independent experiments to determine EC_50_ value. Fold-change: EC_50_ of variant virus containing PA amino acid substitution relative to subtype-specific median or the wild-type PA sequence-matched virus, respectively. Variant viruses lacking PA markers were used to determine the median EC_50_s (Appendix Table 3, https://wwwnc.cdc.gov/EID/article/30/11/24-0892-App1.pdf). Dashes indicate the absence of known or suspected molecular markers for reduced baloxavir susceptibility. EC_50_, 50% effective concentration; IRINA, influenza replication inhibition neuraminidase-based assay; NA, not applicable; PA, polymerase acidic; PA-CEN, PA cap-dependent endonuclease;. †PA-CEN domain encompasses the N-terminal amino acid residues 1 to 209 in PA protein. ‡These viruses were previously tested (*40*). ¶Source: Centers for Disease Control and Prevention Baloxavir Susceptibility Reference Virus Panel (IRR: FR-1678 ver1.1) (https://www.isirv.org/site/index.php/reference-panel).

Overall, sequence analysis results supplemented with those from NA inhibition assay and IRINA suggest that the variant viruses isolated during January 2013–April 2024 maintain susceptibility to NAIs and PA-CENIs. Only 1 of 147 viruses (A/Iowa/33/2017 with PA-I38M) exhibited decreased susceptibility to baloxavir.

### Neutralization by Antibodies Targeting HA

HA is considered an attractive target for development of mAbs as a non–small molecule treatment option (*23*). We next examined the neutralization activity of 2 human mAbs—FI6v3 and CR9114—targeting the HA-stem regions of group 1 and 2 influenza A viruses (*42*,*43*). Using IRINA, we observed a wide range of EC_50_s (0.03 to >10 μg/mL) for both mAbs when tested against a subset of viruses (n = 22) from the 3 subtypes (Table 3). Specifically, we observed broad variation in EC_50_s for A(H1N2)v viruses from clade 1B.2.1 (0.03 to >10 µg/mL for FI6v3 and 0.03–4.02 µg/mL for CR9114) (Table 3). In addition, within each subtype, >1 mAb failed to neutralize viruses even at the highest concentration tested (Table 3). We inspected HA sequences to identify variations in mAb epitopes (*42*,*43*), but none had an apparent effect (Appendix Table 4). Those observations suggest a role of amino acid differences outside the known epitopes. Overall, CR9114 was slightly more effective against the A(H1)v subtype compared with FI6V3.

**Table 3:** Neutralization of variant viruses by broadly reactive human mAbs in IRINA in study of antiviral susceptibility of swine-origin influenza A viruses isolated from humans, United States*

Influenza viruses	HA clade	Mean EC_50_ + SD, µg/mL		HA Acc. ID†
		FI6v3	CR9114	
A(H1N1)v, n = 7				
A/California/07/2009	1A.3.3.2	>10	3.39 + 0.74	EPI1161425
A/North Carolina/01/2021	1A.3.3.2	1.83 + 0.35	0.48 + 0.14	EPI1869574
A/North Dakota/12226/2021	1A.3.3.c-c1	7.91 + 1.33	1.81 + 0.25	EPI1918841
A/Missouri/12/2012	1A.3.3.3-c3	2.15 + 0.15	0.62 + 0.06	EPI395313
A/Arkansas/14/2013	1A.3.3.3-c3	7.79 + 0.35	2.89 + 0.18	EPI471102
A/Arkansas/15/2013	1A.3.3.3-c3	4.52 + 0.94	1.38 + 0.18	EPI482785
A/Wisconsin/03/2021	1A.3.3.3-c3	1.18 + 0.20	0.55 + 0.02	EPI1868840
A(H1N2)v, n = 7				
A/Ohio/24/2017	1A.1.1.3	>10	5.31 + 0.66	EPI1056725
A/Pennsylvania/27/2024	1A.1.1.3	1.46 + 0.06	1.01 + 0.04	EPI3171496
A/Michigan/382/2018	1B.2.1	>10	4.02 + 1.73	EPI1271034
A/Ohio/35/2017	1B.2.1	0.15 + 0.01	0.16 +0.03	EPI1056733
A/Iowa/04/2021	1B.2.1	0.03 + 0.01	0.03 + 0.01	EPI3215541
A/Ohio/28/2022	1B.2.1	3.72 + 0.36	1.33 + 0.06	EPI2193021
A/Michigan/48/2023	1B.2.1	5.75 + 2.24	1.33 + 0.24	EPI2687008
A(H3N2)v, n = 8				
A/Hawaii/28/2020	1990.1	>10	>10	EPI1804523
A/Iowa/04/2013	1990.4.a	5.94 + 1.25	2.26 +1.23	EPI516844
A/Wisconsin/24/2014	1990.4.a	>10	>10	EPI557542
A/Michigan/39/2015	1990.4.a	>10	>10	EPI642513
A/Ohio/02/2014	1990.4.b1	>10	6.59 + 1.37	EPI539159
A/Ohio/27/2016	2010.1	5.82 + 2.38	>10	EPI881739
A/Ohio/13/2017	2010.1	2.30 + 0.54	>10	EPI1056653
A/Wisconsin/01/2021	2010.1	0.64 + 0.11	4.48 + 0.40	EPI1843130
Reference seasonal viruses‡				
A/Illinois/08/2018 (H1N1)pdm09	N/A	8.29 + 1.11	1.69 + 0.07	EPI1259741
A/Louisiana/50/2017 (H3N2)	N/A	6.41 + 1.29	>10	EPI1259757

*Each virus was tested in >3 independent experiments to determine EC_50._ EC_50_, 50% effective concentration; HA, hemagglutinin; IRINA, influenza replication inhibition neuraminidase-based assay; N/A, not applicable. †Accession number of HA sequences deposited to the GISAID database (https://gisaid.org) (accessed on April, 2024). ‡Source: Centers for Disease Control and Prevention antiviral susceptibility reference virus panels (FR-1678 ver1.1) (https://www.isirv.org/site/index.php/reference-panel).

Finally, to determine the antigenic relatedness between the variant viruses and select candidate vaccine viruses (CVVs), we used polyclonal postinfection ferret antiserum in an HI assay and IRINA. We specifically assessed the antigenic relatedness of clade 1B.2.1 (delta 2) A(H1N2)v viruses and A/Ohio/35/2017 (OH/17), a clade-specific CVV (Table 4). Results from both assays indicated that OH/17 antiserum had reduced reactivity to variant viruses (HI 4- to 64-fold; IRINA 9- to 144-fold). We observed the greatest reduction in neutralization activity for 2 viruses, both from 2023 (Table 4), that lack a potential glycosylation motif at residue 89 (H1 numbering) and contain substitutions at antigenic sites Ca (K168T, G237K), Sb (H193N) and 5 additional residues (Appendix Table 5). The OH/17 antiserum reacted somewhat better against the seasonal CVV A/Victoria/2570/2019 (H1N1)pdm09 than to variant viruses from 2023, indicating notable antigenic evolution among swine-origin viruses. Antiserum raised against A/Michigan/48/2023 reacted poorly (HI 8- to 64-fold; IRINA 29-79-fold) against A(H1N2)v viruses collected during 2017–2021 and very poorly (IRINA 134-fold) against A/Victoria/2570/2019 (H1N1)pdm09. As we expected, antiserum raised against A/Victoria/2570/2019 (H1N1)pdm09 produced poor cross-reactivity with A(H1N2)v viruses in both assays (Table 4).

**Table 4:** Antigenic analysis of A(H1N2)v viruses with ferret antiserum using HI and IRINA in study of antiviral susceptibility of swine-origin influenza A viruses isolated from humans, United States*

Influenza viruses	Ferret antiserum, titer (fold change)							
	A/Ohio/35/2017			A/Michigan/48/2023			A/Victoria/2570/2019	
	HI	IRINA		HI	IRINA		HI	IRINA
A(H1N2)v, clade 1B.2.1								
A/Ohio/35/2017, CVV	2,560 (1)	29,041 (1)		20 (64)	228 (79)		<10	<80
Test viruses								
A/Michigan/383/2018	320 (8)	1,131 (26)		80 (16)	469 (38)		<10	<80
A/Iowa/04/2021	640 (4)	3,290 (9)		160 (8)	620 (29)		<10	<80
A/Ohio/28/2022	160 (16)	1,542 (19)		640 (2)	11,677 (2)		<10	<80
A/Montana/28/2023	80 (32)	252 (115)		2,560 (1)	21,399 (1)		<10	<80
A/Michigan/48/2023	40 (64)	201 (144)		1,280 (1)	17,987 (1)		<10	<80
Reference seasonal A(H1N1)pdm09								
A/Victoria/2570/2019, CVV	160 (16)	1,269 (23)		<10	134 (134)		5,120 (1)	81,920 (1)

*HI titer determined using conventional method. IRINA titer determined using curve-fitting, 50% neutralization. Titers are reciprocal of antiserum dilution factor. Underlined values are reactivity titers of antiserum to homologous virus antigens. CVV, candidate vaccine virus; HI, hemagglutination inhibition; IRINA, influenza replication inhibition neuraminidase-based assay.

## Discussion

In this study, we assessed the susceptibility of US influenza variant viruses to influenza antiviral drugs approved by the Food and Drug Administration using a combination of sequence-based analysis and phenotypic testing. In recent years, low proportions (<1%) of seasonal viruses contained mutations that might reduce their susceptibility to antiviral drugs recommended by CDC (*39*,*41*). Our results indicated that the frequency of variant viruses resistant to those antiviral drugs was also low (<1%). As for seasonal viruses, variant viruses collected after the A(H3N2)v virus outbreaks in 2011–2012 were resistant to M2 blockers because of S31N in M2 protein. Whole-genome sequence data supports the conclusion that this resistance was acquired through reverse zoonosis and reassortment (*11*). Conversely, all 2013–2024 variant viruses were deemed susceptible to NAIs because no markers of resistance were identified, and we observed normal inhibition in an NA inhibition assay. Of the known PA substitutions of interest (*33*), only I38M was detected in a single A(H1N1)v virus, which displayed decreased baloxavir susceptibility, as expected.

With the goal of improving interpretation and harmonizing reporting of testing outcomes, we generated subtype-specific baseline antiviral susceptibilities of variant viruses. In the NA inhibition assay, 1 flagged virus with S247N displayed up to 4-fold increase in IC_50_ for NAIs regardless of the comparator. This finding is similar to other reports and was interpreted as normal inhibition (*35*,*44*,*45*). However, S247N in a clade 2.3.4 A(H5N1) virus was associated with reduced inhibition by oseltamivir (*34*). Another A(H1N1)v virus, A/Missouri/12/2012, had I427V substitution. Its effect on oseltamivir susceptibility remains unknown because a sequence-matched control was unavailable for testing. However, I427V would be reported as conferring reduced inhibition (17-fold) by oseltamivir when comparison is done using the A(H1N1)v subtype-specific baseline but not for the N1-classical virus baseline (8-fold). Those results underscore the uncertainties in the current interpretation of NA inhibition data and a need to refine the WHO Global Influenza Surveillance and Research System reporting criteria for zoonotic viruses.

In addition to assessing NAI susceptibility, we performed phenotypic testing with baloxavir using IRINA, a new assay developed to improve antiviral phenotyping throughput and turnaround time (*30*). In this assay, I38M in A(H1N1)v virus conferred decreased susceptibility to baloxavir compared with either sequence-matched control or the subtype-specific baseline. In seasonal viruses, E199G confers a variable effect (1- to 7-fold) on EC_50_ (*33*,*46*). In this study, E199G did not alter baloxavir susceptibility of an A(H3N2)v virus when compared in a similar manner, indicating the role of a virus’s genetic background.

While establishing the baloxavir susceptibility baseline, we noticed that A(H1N1)v viruses appeared to be slightly more susceptible, even when they shared an identical PA-CEN domain sequence with viruses from the 2 other subtypes. Baloxavir exerts its antiviral effect by inhibiting synthesis of viral mRNA, thus preventing synthesis of viral proteins. In IRINA, baloxavir and virus are added to cells simultaneously, and viral replication is limited to a single cycle because of the absence of TPCK-trypsin in media. Under such conditions, viruses displaying slower binding and internalization might appear to be slightly more susceptible to baloxavir. Therefore, when interpreting baloxavir EC_50_ of variant viruses, using a subtype-specific baseline might be prudent, especially considering the rather low provisional cutoff (3-fold) for reporting reduced drug susceptibility. Alternatively, the cutoff could be adjusted to a greater value (e.g., >5-fold over baseline) to prevent overreporting decreased susceptibility, such as for A/Michigan/288/2019 (H1N1)v, which exceeded the 3-fold threshold for the A(H1N1)v subtype, despite lacking PA-CEN substitutions.

IRINA was developed to test viruses against antiviral drugs with different mechanisms of action (*30*). In this study, mAbs targeting the HA stem region, FI6V3 and CR9114, demonstrated broad activity. However, 1 or both mAbs failed to neutralize one third of variant viruses. Variations in the mapped antigenic epitopes were observed, but they could not explain the observed neutralization patterns, indicating an involvement of residues outside of the identified epitopes. Of note, IRINA measures direct neutralization, and HA-targeting antibodies could possibly produce antiviral effects through alternative mechanisms when used in vivo (e.g., antibody effector functions) (*30*).

We used IRINA alongside HI assay to antigenically characterize A(H1N2)v viruses from clade 1B.2.1. As expected, IRINA titers were greater than those determined in HI assay because only a portion of neutralizing antibodies can prevent agglutination of erythrocytes. Moreover, IRINA titers are determined using curve-fitting and IC_50_-based calculations. Despite differences in absolute titers between IRINA and HI, the fold changes in both assays indicated similar reactivity patterns. Moreover, antiserum raised against OH/17 (CVV for clade 1B.2.1), poorly neutralized viruses from the same clade collected in 2023, which indicated antigenic divergence. Taken together, the IRINA and HI data underscore the need to closely monitor antigenic properties of variant viruses and to promptly update CVVs as part of pandemic preparedness.

Although sequencing information is indispensable for risk assessment, sequence-only analysis might fail to predict the effect of mutations and their combinations on antiviral susceptibility as it could be specific to virus type, subtype, clade, or strain (*31*–*33*). Nonetheless, sequence-only data can be used to generate recombinant NA proteins (*15*) or reverse genetics-derived viruses to conduct antiviral testing (*35*).

The first limitation of our study is that not all variant viruses collected since 2013 were subjected to phenotypic testing. Therefore, we cannot rule out that few untested viruses with rare mutations would exhibit decreased drug susceptibility. In addition, interpreting laboratory phenotypic data is challenging because of the lack of laboratory correlates for clinically relevant antiviral resistance, even for the commonly used oseltamivir. Last, antiviral susceptibility testing is done using in-house–developed assays, which are known to produce different IC_50_/EC_50_ results. Criteria used to report viruses that are potentially resistant are arbitrarily based on fold change in IC_50_/EC_50_ values, which are assay dependent (*31*).

Variant viruses have been detected and characterized in many countries, including recently from Brazil, Spain, and the United Kingdom (*47*,*48*). The genetic makeup differs in swine viruses from different parts of the world, which might manifest in different susceptibilities to antiviral drugs. Laboratories that are unable to establish baseline susceptibility for variant viruses using their phenotypic assays may benefit from including well-characterized reference seasonal influenza A viruses when testing newly emerged viruses. This practice would help harmonize testing methodologies and interpretation of laboratory data to improve our knowledge of the viruses that continuously pose a pandemic threat.

Appendix Additional information about antiviral susceptibility of swine-origin influenza A viruses isolated from humans, United States
