# Supplementary material for: Antiviral Susceptibility of Swine-Origin Influenza A Viruses Isolated from Humans, United States
Source: Emerg Infect Dis. 2024 Nov;30(11):2303–12. doi: 10.3201/eid3011.240892 (PMC11521183; doi:10.3201/eid3011.240892)
Supplement: Appendix — Additional information about antiviral susceptibility of swine-origin influenza A viruses isolated from humans, United States [file 24-0892-Techapp-s1.pdf]

Article DOI: https://doi.org/10.3201/eid3011.240892

EID cannot ensure accessibility for Supplemental Materials supplied by authors. Readers who have difficulty accessing supplementary content should contact the authors for assistance.

Antiviral Susceptibility of Swine-Origin Influenza A Viruses Isolated from Humans, United States

Appendix.

Appendix Table 1. Variant influenza viruses collected in the United States between January 2013 and April 2024*

	Virus gene segments†									
	PA			NA			M			
Variant virus name	GISAID ID	Lineage‡	Change	GISAID ID	Lineage‡	Change	GISAID ID	Lineage‡	M2 Change	Originating Lab and Submitter Institution Name
A(H1N1)v (n = 19)										
**A/Arkansas/14/2013**	EPI471101	pdm09	-	EPI471104	Classical	-	EPI471105	pdm09	S31N	Arkansas Department of Health/CDC-Atlanta
**A/Arkansas/15/2013**	EPI482781	pdm09	-	EPI482784	Classical	-	EPI482780	pdm09	S31N	Arkansas Department of Health/CDC-Atlanta
**A/Minnesota/33/2014**	EPI558118	TRIG	-	EPI558120	Classical	-	EPI558117	pdm09	S31N	Minnesota Department of Health/CDC-Atlanta
**A/Ohio/09/2015**	EPI590120	TRIG	-	EPI590123	Classical	-	EPI590119	pdm09	S31N	Ohio Department of Health Laboratories/CDC-Atlanta
**A/Iowa/39/2015**	EPI638966	pdm09	-	EPI638969	Classical	-	EPI638965	pdm09	S31N	Iowa State Hygienic Laboratory/CDC-Atlanta
A/Minnesota/46/2015	EPI677673	pdm09	-	EPI677676	Classical	-	EPI677672	pdm09	S31N	Minnesota Department of Health/CDC-Atlanta
**A/Iowa/33/2017§**	EPI1311341	pdm09	I38M	EPI1311344	pdm09	-	EPI1311340	pdm09	S31N	Iowa State Hygienic Laboratory/CDC-Atlanta
**A/Michigan/288/2019¶**	EPI1565951	LAIV	-	EPI1565954	pdm09	-	EPI1565950	pdm09	S31N	Michigan Department of Community Health/CDC-Atlanta
**A/Iowa/22/2020#**	EPI1840422	pdm09	-	EPI1840425	pdm09	-	EPI1840421	pdm09	S31N	Iowa State Hygienic Laboratory/CDC-Atlanta
**A/Iowa/23/2020#**	EPI2020649	pdm09	-	EPI2020652	pdm09	-	EPI2020648	pdm09	S31N	Iowa State Hygienic Laboratory/CDC-Atlanta
A/North Carolina/15/2020	EPI1853334	TRIG	-	EPI1853337	Classical	-	EPI1853333	pdm09	S31N	North Carolina State Laboratory of Public Health/CDC-Atlanta
**A/Iowa/01/2021#**	EPI1856079	pdm09	-	EPI1856082	pdm09	-	EPI1856078	pdm09	S31N	Iowa State Hygienic Laboratory/CDC-Atlanta
**A/Iowa/02/2021**	EPI1868844	pdm09	-	EPI1868846	pdm09	S247N	EPI1868843	pdm09	S31N	Iowa State Hygienic Laboratory/CDC-Atlanta
A/Iowa/05/2021	EPI1941462	TRIG	-	EPI1941465	pdm09	-	EPI1941461	pdm09	S31N	Iowa State Hygienic Laboratory/CDC-Atlanta
A/Iowa/06/2021	EPI1941478	TRIG	-	EPI1941481	pdm09	-	EPI1941477	pdm09	S31N	Iowa State Hygienic Laboratory/CDC-Atlanta
**A/North Carolina/01/2021#**	EPI2319606	pdm09	-	EPI2319609	pdm09	-	EPI2319605	pdm09	S31N	North Carolina State Laboratory of Public Health/CDC-Atlanta
**A/North Dakota/12226/2021**	EPI1918837	TRIG	-	EPI1918840	pdm09	-	EPI1918836	pdm09	S31N	U.S. Air Force School of Aerospace Medicine /
**A/Wisconsin/03/2021**	EPI1868836	pdm09	-	EPI1868839	Classical	-	EPI1868835	pdm09	S31N	Wisconsin State Laboratory of Hygiene/CDC-Atlanta
A/Wisconsin/04/2021	EPI1927634	pdm09	-	EPI1927637	Classical	-	EPI1927638	pdm09	S31N	Wisconsin State Laboratory of Hygiene/CDC-Atlanta
A(H1N2)v (n = 22)										
A/Iowa/32/2016	EPI864023	TRIG	-	EPI864026	2002	-	EPI864022	pdm09	S31N	Iowa State Hygienic Laboratory/CDC-Atlanta
A/Colorado/16/2017	EPI1312175	TRIG	-	EPI1312178	2002	-	EPI1312174	pdm09	S31N	Colorado Department of Health Lab/CDC-Atlanta
**A/Ohio/24/2017**	EPI1056721	pdm09	-	EPI1056724	2002	-	EPI1056720	pdm09	S31N	Ohio Department of Health Laboratories/CDC-Atlanta
**A/Ohio/35/2017**	EPI1056729	TRIG	-	EPI1056732	2002	-	EPI1056728	pdm09	S31N	Ohio Department of Health Laboratories/CDC-Atlanta
A/California/58/2018	EPI1271054	TRIG	-	EPI1271057	1998	-	EPI1271053	pdm09	S31N	California Department of Health Services/CDC-Atlanta

	Virus gene segments†									
	PA			NA			M			
Variant virus name	GISAID ID	Lineage‡	Change	GISAID ID	Lineage‡	Change	GISAID ID	Lineage‡	M2 Change	Originating Lab and Submitter Institution Name
**A/California/62/2018**	EPI1311357	TRIG	-	EPI1311360	1998	-	EPI1311356	pdm09	S31N	California Department of Health Services/CDC-Atlanta
**A/California/63/2018**	EPI1311365	TRIG	-	EPI1311368	1998	-	EPI1311364	pdm09	S31N	California Department of Health Services/CDC-Atlanta
**A/Michigan/382/2018**	EPI1271030	TRIG	-	EPI1271033	1998	-	EPI1271029	pdm09	S31N	Michigan Department of Community Health/CDC-Atlanta
**A/Michigan/383/2018**	EPI1271062	TRIG	-	EPI1271065	1998	-	EPI1271061	pdm09	S31N	Michigan Department of Community Health/CDC-Atlanta
**A/Michigan/384/2018**	EPI1271038	TRIG	-	EPI1271041	1998	-	EPI1271037	pdm09	S31N	Michigan Department of Community Health/CDC-Atlanta
**A/Ohio/25/2018**	EPI1311349	TRIG	-	EPI1311352	1998	-	EPI1311348	pdm09	S31N	Ohio Department of Health Laboratories/CDC-Atlanta
A/Ohio/28/2018	EPI1311373	TRIG	-	EPI1311376	1998	-	EPI1311372	pdm09	S31N	Ohio Department of Health Laboratories/CDC-Atlanta
A/California/71/2021	EPI1994317	pdm09	-	EPI1994320	2002	-	EPI1994316	pdm09	S31N	California Department of Health Services/CDC-Atlanta
**A/Iowa/04/2021**	EPI3215537	TRIG	-	EPI3215540	1998	-	EPI3215536	pdm09	S31N	Iowa State Hygienic Laboratory/CDC-Atlanta
A/Georgia/08/2022	EPI2193041	TRIG	-	EPI2193044	1998	-	EPI2193040	pdm09	S31N	Georgia Public Health Laboratory/CDC-Atlanta
A/Michigan/42/2022	EPI2193025	TRIG	-	EPI2193028	2002	-	EPI2193024	pdm09	S31N	Michigan Department of Community Health/CDC-Atlanta
**A/Ohio/28/2022**	EPI2193017	TRIG	-	EPI2193020	1998	-	EPI2193016	pdm09	S31N	Ohio Department of Health Laboratories/CDC-Atlanta
A/Oregon/32/2022	EPI2134166	TRIG	-	EPI2134169	1998	-	EPI2134165	pdm09	S31N	Oregon Public Health Laboratory/CDC-Atlanta
A/Wisconsin/51/2022	EPI2193033	TRIG	-	EPI2193036	1998	-	EPI2193032	pdm09	S31N	Wisconsin State Laboratory of Hygiene/CDC-Atlanta
**A/Michigan/48/2023**	EPI2687004	TRIG	-	EPI2687007	1998	-	EPI2687003	pdm09	S31N	Michigan Department of Community Health/CDC-Atlanta
**A/Montana/28/2023**	EPI3215545	TRIG	-	EPI3215548	1998	-	EPI3215544	pdm09	S31N	Montana Public Health Laboratory/CDC-Atlanta
**A/Pennsylvania/27/2024**	EPI3171495	TRIG	-	EPI3171494	1998	-	EPI3171501	pdm09	S31N	Pennsylvania Department of Health/CDC-Atlanta
A(H3N2)v (n = 106)										
A/Ohio/05/2013	EPI464866	pdm09	-	EPI464869	2002	-	EPI464865	pdm09	S31N	Ohio Department of Health Laboratories/CDC-Atlanta
A/Illinois/03/203	EPI465361	pdm09	-	EPI465364	2002	-	EPI465360	pdm09	S31N	Illinois Department of Public Health-Chicago/CDC-Atlanta
A/Indiana/04/2013	n/a	n/a	n/a	EPI464501	2002	-	EPI464502	pdm09	S31N	Indiana State Department of Health Laboratories/CDC-Atlanta
A/Indiana/05/2013	n/a	n/a	n/a	EPI464498	2002	-	EPI464499	pdm09	S31N	Indiana State Department of Health Laboratories/CDC-Atlanta
A/Indiana/07/2013	n/a	n/a	n/a	EPI465383	2002	-	EPI465384	pdm09	S31N	Indiana State Department of Health Laboratories/CDC-Atlanta
A/Indiana/08/2013	n/a	n/a	n/a	EPI461879	2002	-	EPI461878	pdm09	S31N	Indiana State Department of Health Laboratories/CDC-Atlanta
A/Indiana/09/2013	EPI461882	TRIG	-	EPI461885	2002	-	EPI461886	pdm09	S31N	Indiana State Department of Health Laboratories/CDC-Atlanta
A/Indiana/10/2013	EPI464507	TRIG	-	EPI464510	2002	-	EPI464506	pdm09	S31N	Indiana State Department of Health Laboratories/CDC-Atlanta
A/Indiana/11/2013	n/a	n/a	n/a	EPI482812	2002	-	EPI482813	pdm09	S31N	Indiana State Department of Health Laboratories/CDC-Atlanta
A/Indiana/14/2013	n/a	n/a	n/a	EPI462220	2002	-	EPI462219	pdm09	S31N	Indiana State Department of Health Laboratories/CDC-Atlanta
A/Indiana/15/2013	n/a	n/a	n/a	EPI462229	2002	-	EPI462228	pdm09	S31N	Indiana State Department of Health Laboratories/CDC-Atlanta
A/Indiana/16/2013	n/a	n/a	n/a	EPI462217	2002	-	EPI462216	pdm09	S31N	Indiana State Department of Health Laboratories/CDC-Atlanta
A/Indiana/17/2013	n/a	n/a	n/a	EPI465377	2002	-	EPI465378	pdm09	S31N	Indiana State Department of Health Laboratories/CDC-Atlanta
A/Indiana/18/2013	n/a	n/a	n/a	EPI462226	2002	-	EPI462225	pdm09	S31N	Indiana State Department of Health Laboratories/CDC-Atlanta
A/Indiana/19/2013	n/a	n/a	n/a	EPI462222	2002	-	EPI462223	pdm09	S31N	Indiana State Department of Health Laboratories/CDC-Atlanta
A/Indiana/21/2013	n/a	n/a	n/a	EPI482815	2002	-	EPI482816	pdm09	S31N	Indiana State Department of Health Laboratories/CDC-Atlanta
**A/Iowa/04/2013**	EPI516840	pdm09	-	EPI516843	2002	-	EPI516839	pdm09	S31N	Iowa State Hygienic Laboratory/CDC-Atlanta
**A/Ohio/05/2013**	EPI464866	pdm09	-	EPI464869	2002	-	EPI464865	pdm09	S31N	Ohio Department of Health Laboratories/CDC-Atlanta
**A/Ohio/02/2014**	EPI539158	TRIG	-	EPI539161	2002	-	EPI539162	pdm09	S31N	Ohio Department of Health Laboratories/CDC-Atlanta
**A/Ohio/4319/2014**	EPI541959	TRIG	-	EPI541962	2002	-	EPI541963	pdm09	S31N	U.S. Air Force School of Aerospace Medicine/CDC-Atlanta
**A/Wisconsin/24/2014**	EPI557538	TRIG	-	EPI557541	2002	-	EPI557537	pdm09	S31N	Wisconsin State Laboratory of Hygiene/CDC-Atlanta
**A/Michigan/39/2015**	EPI642509	TRIG	-	EPI642512	2002	-	EPI642508	pdm09	S31N	Michigan Department of Community Health/CDC-Atlanta
A/Minnesota/38/2015	EPI625704	TRIG	-	EPI625707	2002	-	EPI625703	pdm09	S31N	Minnesota Department of Health/CDC-Atlanta
**A/New Jersey/53/2015**	EPI685730	TRIG	E199G	EPI685733	1998	-	EPI685729	pdm09	S31N	New Jersey Department of Health & Senior Services/CDC-Atlanta
A/Michigan/82/2016	EPI824761	TRIG	-	EPI824764	2002	-	EPI824760	pdm09	S31N	Michigan Department of Community Health/CDC-Atlanta

	Virus gene segments†									
	PA			NA			M			
Variant virus name	GISAID ID	Lineage‡	Change	GISAID ID	Lineage‡	Change	GISAID ID	Lineage‡	M2 Change	Originating Lab and Submitter Institution Name
**A/Michigan/83/2016**	EPI824771	TRIG	-	EPI824774	2002	-	EPI824770	pdm09	S31N	Michigan Department of Community Health/CDC-Atlanta
**A/Michigan/84/2016**	EPI838247	TRIG	-	EPI824766	2002	-	EPI838246	pdm09	S31N	Michigan Department of Community Health/CDC-Atlanta
A/Michigan/87/2016	EPI881758	TRIG	-	EPI838250	2002	-	EPI881757	pdm09	S31N	Michigan Department of Community Health/CDC-Atlanta
A/Michigan/88/2016	EPI881699	TRIG	-	EPI881698	2002	-	EPI838252	pdm09	S31N	Michigan Department of Community Health/CDC-Atlanta
A/Michigan/89/2016	EPI881709	TRIG	-	EPI838265	2002	-	EPI881708	pdm09	S31N	Michigan Department of Community Health/CDC-Atlanta
A/Michigan/90/2016	EPI838260	TRIG	-	EPI838263	2002	-	EPI838259	pdm09	S31N	Michigan Department of Community Health/CDC-Atlanta
A/Michigan/93/2016	EPI838271	TRIG	-	EPI838274	2002	-	EPI838270	pdm09	S31N	Michigan Department of Community Health/CDC-Atlanta
A/Michigan/94/2016	EPI838278	TRIG	-	EPI838281	2002	-	EPI838277	pdm09	S31N	Michigan Department of Community Health/CDC-Atlanta
A/Michigan/95/2016	n/a	n/a	n/a	EPI838283	2002	-	n/a	n/a	n/a	Michigan Department of Community Health/CDC-Atlanta
A/Michigan/96/2016	EPI881717	TRIG	-	EPI838285	2002	-	EPI881716	pdm09	S31N	Michigan Department of Community Health/CDC-Atlanta
**A/Ohio/27/2016**	EPI881735	TRIG	-	EPI881738	2002	-	EPI881734	pdm09	S31N	Ohio Department of Health Laboratories/CDC-Atlanta
**A/Ohio/28/2016**	EPI824753	TRIG	-	EPI824756	2002	-	EPI824752	pdm09	S31N	Ohio Department of Health Laboratories/CDC-Atlanta
A/Ohio/29/2016	EPI881704	TRIG	-	EPI838254	2002	-	EPI881703	pdm09	S31N	Ohio Department of Health Laboratories/CDC-Atlanta
A/Ohio/32/2016	EPI881723	TRIG	-	EPI838287	2002	-	EPI881722	pdm09	S31N	Ohio Department of Health Laboratories/CDC-Atlanta
A/Ohio/33/2016	n/a	n/a	n/a	EPI838267	2002	-	n/a	n/a	n/a	Ohio Department of Health Laboratories/CDC-Atlanta
A/Ohio/35/2016	EPI881729	TRIG	-	EPI838289	2002	-	EPI881728	pdm09	S31N	Ohio Department of Health Laboratories/CDC-Atlanta
A/Delaware/33/2017	EPI1311389	TRIG	-	EPI1311392	2002	-	EPI1311388	pdm09	S31N	Delaware Public Health Lab/CDC-Atlanta
A/District of Columbia/01/2017	EPI1311445	TRIG	-	EPI1311448	2002	-	EPI1311444	pdm09	S31N	Maryland Department of Health and Mental Hygiene/CDC-Atlanta
A/District of Columbia/02/2017	EPI1311437	TRIG	-	EPI1311440	2002	-	EPI1311436	pdm09	S31N	Maryland Department of Health and Mental Hygiene/CDC-Atlanta
A/Iowa/38/2017	EPI1311381	TRIG	-	EPI1311384	2002	-	EPI1311380	pdm09	S31N	Iowa State Hygienic Laboratory/CDC-Atlanta
A/Maryland/42/2017	EPI1154896	TRIG	-	EPI1154899	2002	-	EPI1154895	pdm09	S31N	Maryland Department of Health and Mental Hygiene/CDC-Atlanta
A/Maryland/43/2017	EPI1154904	TRIG	-	EPI1154907	2002	-	EPI1154903	pdm09	S31N	Maryland Department of Health and Mental Hygiene/CDC-Atlanta
A/Maryland/44/2017	EPI1154912	TRIG	-	EPI1154915	2002	-	EPI1154911	pdm09	S31N	Maryland Department of Health and Mental Hygiene/CDC-Atlanta
A/Maryland/45/2017	EPI1154920	TRIG	-	EPI1154923	2002	-	EPI1154919	pdm09	S31N	Maryland Department of Health and Mental Hygiene/CDC-Atlanta
A/Maryland/46/2017	EPI1154928	TRIG	-	EPI1154931	2002	-	EPI1154927	pdm09	S31N	Maryland Department of Health and Mental Hygiene/CDC-Atlanta
A/Maryland/49/2017	EPI1154936	TRIG	-	EPI1154939	2002	-	EPI1154935	pdm09	S31N	Maryland Department of Health and Mental Hygiene/CDC-Atlanta
A/Maryland/50/2017	EPI1154944	TRIG	-	EPI1154947	2002	-	EPI1154943	pdm09	S31N	Maryland Department of Health and Mental Hygiene/CDC-Atlanta
A/Maryland/51/2017	EPI1154952	TRIG	-	EPI1154955	2002	-	EPI1154951	pdm09	S31N	Maryland Department of Health and Mental Hygiene/CDC-Atlanta
A/Maryland/52/2017	EPI1154960	TRIG	-	EPI1154963	2002	-	EPI1154959	pdm09	S31N	Maryland Department of Health and Mental Hygiene/CDC-Atlanta
A/Maryland/54/2017	EPI1154968	TRIG	-	EPI1154971	2002	-	EPI1154967	pdm09	S31N	Maryland Department of Health and Mental Hygiene/CDC-Atlanta
A/Maryland/55/2017	EPI1154976	TRIG	-	EPI1154979	2002	-	EPI1154975	pdm09	S31N	Maryland Department of Health and Mental Hygiene/CDC-Atlanta
A/Maryland/57/2017	n/a	n/a	n/a	EPI1154981	2002	-	n/a	n/a	n/a	Maryland Department of Health and Mental Hygiene/CDC-Atlanta
A/Maryland/58/2017	EPI1154986	TRIG	-	EPI1154989	2002	-	EPI1154985	pdm09	S31N	Maryland Department of Health and Mental Hygiene/CDC-Atlanta
A/Maryland/59/2017	EPI1154994	TRIG	-	EPI1154997	2002	-	EPI1154993	pdm09	S31N	Maryland Department of Health and Mental Hygiene/CDC-Atlanta
A/Maryland/60/2017	EPI1155002	TRIG	-	EPI1155005	2002	-	EPI1155001	pdm09	S31N	Maryland Department of Health and Mental Hygiene/CDC-Atlanta
A/Maryland/61/2017	EPI1155010	TRIG	-	EPI1155013	2002	-	EPI1155009	pdm09	S31N	Maryland Department of Health and Mental Hygiene/CDC-Atlanta
A/Maryland/62/2017	EPI1155018	TRIG	-	EPI1155021	2002	-	EPI1155017	pdm09	S31N	Maryland Department of Health and Mental Hygiene/CDC-Atlanta
A/Maryland/63/2017	EPI1155026	TRIG	-	EPI1155029	2002	-	EPI1155025	pdm09	S31N	Maryland Department of Health and Mental Hygiene/CDC-Atlanta
A/Maryland/64/2017	EPI1155034	TRIG	-	EPI1155037	2002	-	EPI1155033	pdm09	S31N	Maryland Department of Health and Mental Hygiene/CDC-Atlanta
A/Maryland/65/2017	EPI1155042	TRIG	-	EPI1155045	2002	-	EPI1155041	pdm09	S31N	Maryland Department of Health and Mental Hygiene/CDC-Atlanta
A/Maryland/66/2017	n/a	n/a	n/a	EPI1155052	2002	-	EPI1155049	pdm09	S31N	Maryland Department of Health and Mental Hygiene/CDC-Atlanta
A/Maryland/67/2017	EPI1155057	TRIG	-	EPI1155060	2002	-	EPI1155056	pdm09	S31N	Maryland Department of Health and Mental Hygiene/CDC-Atlanta
A/Maryland/68/2017	n/a	n/a	n/a	EPI1203837	2002	-	EPI1203835	pdm09	S31N	Maryland Department of Health and Mental Hygiene/CDC-Atlanta

	Virus gene segments†									
	PA			NA			M			
Variant virus name	GISAID ID	Lineage‡	Change	GISAID ID	Lineage‡	Change	GISAID ID	Lineage‡	M2 Change	Originating Lab and Submitter Institution Name
A/Maryland/69/2017	EPI1155065	TRIG	-	EPI1155068	2002	-	EPI1155064	pdm09	S31N	Maryland Department of Health and Mental Hygiene/CDC-Atlanta
A/Maryland/70/2017	EPI1155073	TRIG	-	EPI1155076	2002	-	EPI1155072	pdm09	S31N	Maryland Department of Health and Mental Hygiene/CDC-Atlanta
A/Maryland/71/2017	EPI1155081	TRIG	-	EPI1155084	2002	-	EPI1155080	pdm09	S31N	Maryland Department of Health and Mental Hygiene/CDC-Atlanta
A/Maryland/72/2017	EPI1155089	TRIG	-	EPI1155092	2002	-	EPI1155088	pdm09	S31N	Maryland Department of Health and Mental Hygiene/CDC-Atlanta
A/Maryland/73/2017	EPI1155097	TRIG	-	EPI1155100	2002	-	EPI1155096	pdm09	S31N	Maryland Department of Health and Mental Hygiene/CDC-Atlanta
A/Maryland/74/2017	EPI1155105	TRIG	-	EPI1155108	2002	-	EPI1155104	pdm09	S31N	Maryland Department of Health and Mental Hygiene/CDC-Atlanta
A/Maryland/75/2017	EPI1155113	TRIG	-	EPI1155116	2002	-	EPI1155112	pdm09	S31N	Maryland Department of Health and Mental Hygiene/CDC-Atlanta
A/Maryland/76/2017	EPI1155121	TRIG	-	EPI1155124	2002	-	EPI1155120	pdm09	S31N	Maryland Department of Health and Mental Hygiene/CDC-Atlanta
A/Maryland/77/2017	EPI1155129	TRIG	-	EPI1155132	2002	-	EPI1155128	pdm09	S31N	Maryland Department of Health and Mental Hygiene/CDC-Atlanta
A/Maryland/78/2017	EPI1155137	TRIG	-	EPI1155141	2002	-	EPI1155136	pdm09	S31N	Maryland Department of Health and Mental Hygiene/CDC-Atlanta
A/Maryland/79/2017	EPI1155148	TRIG	-	EPI1155152	2002	-	EPI1155147	pdm09	S31N	Maryland Department of Health and Mental Hygiene/CDC-Atlanta
A/Maryland/87/2017	EPI1155157	TRIG	-	EPI1155160	2002	-	EPI1155156	pdm09	S31N	Maryland Department of Health and Mental Hygiene/CDC-Atlanta
A/Michigan/100/2017	EPI1115623	human H3	-	EPI1115626	2002	-	EPI1115622	pdm09	S31N	University of Michigan SPH EPID/CDC-Atlanta
A/Nebraska/11/2017	EPI1311453	TRIG	-	EPI1311456	2002	-	EPI1311452	pdm09	S31N	Washington State Public Health Laboratory/CDC-Atlanta
**A/North Dakota/19/2017**	EPI1311461	TRIG	-	EPI1311464	2002	-	EPI1311460	pdm09	S31N	North Dakota Department of Health/CDC-Atlanta
**A/Ohio/13/2017**	EPI1056649	TRIG	-	EPI1056652	2002	-	EPI1056648	pdm09	S31N	Ohio Department of Health Laboratories/CDC-Atlanta
**A/Ohio/14/2017**	EPI1056657	TRIG	-	EPI1056660	2002	-	EPI1056656	pdm09	S31N	Ohio Department of Health Laboratories/CDC-Atlanta
**A/Ohio/15/2017**	EPI1056665	TRIG	-	EPI1056668	2002	-	EPI1056664	pdm09	S31N	Ohio Department of Health Laboratories/CDC-Atlanta
A/Ohio/17/2017	EPI1056673	TRIG	-	EPI1056676	2002	-	EPI1056672	pdm09	S31N	Ohio Department of Health Laboratories/CDC-Atlanta
A/Ohio/18/2017	EPI1311429	TRIG	-	EPI1311432	2002	-	EPI1311428	pdm09	S31N	Ohio Department of Health Laboratories/CDC-Atlanta
A/Ohio/19/2017	EPI1056681	TRIG	-	EPI1056684	2002	-	EPI1056680	pdm09	S31N	Ohio Department of Health Laboratories/CDC-Atlanta
A/Ohio/20/2017	EPI1056689	TRIG	-	EPI1056692	2002	-	EPI1056688	pdm09	S31N	Ohio Department of Health Laboratories/CDC-Atlanta
A/Ohio/21/2017	EPI1056697	TRIG	-	EPI1056700	2002	-	EPI1056696	pdm09	S31N	Ohio Department of Health Laboratories/CDC-Atlanta
A/Ohio/22/2017	EPI1056705	TRIG	-	EPI1056708	2002	-	EPI1056704	pdm09	S31N	Ohio Department of Health Laboratories/CDC-Atlanta
A/Ohio/23/2017	EPI1056713	TRIG	-	EPI1056716	2002	-	EPI1056712	pdm09	S31N	Ohio Department of Health Laboratories/CDC-Atlanta
A/Ohio/26/2017	EPI1311421	TRIG	-	EPI1311424	2002	-	EPI1311420	pdm09	S31N	Ohio Department of Health Laboratories/CDC-Atlanta
A/Ohio/27/2017	EPI1311397	TRIG	-	EPI1311400	2002	-	EPI1311396	pdm09	S31N	Ohio Department of Health Laboratories/CDC-Atlanta
A/Ohio/28/2017	EPI1311413	TRIG	-	EPI1311416	2002	-	EPI1311412	pdm09	S31N	Ohio Department of Health Laboratories/CDC-Atlanta
**A/Ohio/29/2017**	EPI1311405	TRIG	-	EPI1311408	2002	-	EPI1311404	pdm09	S31N	Ohio Department of Health Laboratories/CDC-Atlanta
A/Texas/105/2017	EPI1010373	TRIG	-	EPI1010376	2002	-	EPI1010372	pdm09	S31N	U.S. Air Force School of Aerospace Medicine/CDC-Atlanta
A/Indiana/27/2018	EPI1272684	TRIG	-	EPI1272687	2002	-	EPI1272683	pdm09	S31N	Indiana State Department of Health Laboratories/CDC-Atlanta
**A/Hawaii/28/2020¶**	EPI1804519	LAIV	-	EPI1804522	1998	-	EPI1804518	LAIV	-	State of Hawaii Department of Health/CDC-Atlanta
**A/Wisconsin/01/2021**	EPI1843126	TRIG	-	EPI1843129	2002	-	EPI1843125	pdm09	S31N	Wisconsin State Laboratory of Hygiene/CDC-Atlanta
A/Michigan/48/2022	EPI2196508	TRIG	-	EPI2196511	2002	-	EPI2196507	pdm09	S31N	Michigan Department of Community Health/CDC-Atlanta
A/New Mexico/19/2022	EPI2201447	TRIG	-	EPI2201450	2002	-	EPI2201446	pdm09	S31N	New Mexico Department of Health/CDC-Atlanta
**A/West Virginia/24/2022**	EPI2134142	TRIG	-	EPI2134145	2002	-	EPI2134141	pdm09	S31N	West Virginia Office of Laboratory Services/CDC-Atlanta
**A/West Virginia/25/2022**	EPI2134150	TRIG	-	EPI2134153	2002	-	EPI2134149	pdm09	S31N	West Virginia Office of Laboratory Services/CDC-Atlanta
**A/West Virginia/26/2022**	EPI2134158	TRIG	-	EPI2134161	2002	-	EPI2134157	pdm09	S31N	West Virginia Office of Laboratory Services/CDC-Atlanta

*Viruses in **boldface** text were used for phenotypic characterization in this study. CDC, Centers for Disease Control and Prevention; M, matrix; n/a, not available; NA, neuraminidase; PA, polymerase acidic; TRIG, triple-reassortment internal gene.

†PA, NA, and M gene segments of 147 swine-origin (variant) influenza A viruses collected in the United States from January 2013 to April 2024 available for analysis and deposited in the Global Initiative on Sharing All Influenza Data (GISAID) database (accessed on April 2024). Sequences were compiled and analyzed using MAFFT version 7 program. Dash lines indicate the absence of known or suspected molecular markers associated with reduce antiviral susceptibility.

‡Lineage, gene segment lineages: TRIG, triple-reassortment internal gene lineage; pdm09, A(H1N1)pdm09 lineage; human H3, seasonal human-like A(H3N2) lineage; LAIV, swine live-attenuated influenza vaccine virus lineage; Classical, N1-classical lineage; 1998, N2–1998 lineage; 2002, N2–2002 lineage.

§Clinical specimen and isolate for A/Iowa/33/2017 (H1N1)v virus contained I38M/I mixture in PA and the two virus populations were separated by biologic cloning (clones 1 and 2, see also Appendix Table 2) (*1*).

¶Variant viruses which contain some segments derived from the A(H3N2) component of a swine LAIV.

	Virus gene segments†									
	PA			NA			M			
Variant virus name	GISAID ID	Lineage‡	Change	GISAID ID	Lineage‡	Change	GISAID ID	Lineage‡	M2 Change	Originating Lab and Submitter Institution Name

#Seasonal A(H1N1)pdm09 viruses that were re-classified as variants (*2*).

Appendix Table 2. NA inhibitor susceptibility of variant influenza viruses assessed by subtype using a fluorescence-based NA inhibition assay*

Influenza A virus	NA lineage	IC_{50} (nM)±SD (fold-change)			
		Zanamivir	Oseltamivir	Peramivir	Laninamivir
Median IC_{50}, A(H1N1)v (n = 15)†		*0.21*	*0.43*	*0.07*	*0.22*
A/Texas/14/2008	N1-Classical	0.21 ± 0.01 (1)	1.03 ± 0.19 (2)	0.10 ± 0.01 (1)	0.31 ± 0.04 (1)
A/California/07/2009	N1-pdm09	0.17 ± 0.02 (1)	0.35 ± 0.05 (1)	0.06 ± 0.01 (1)	0.16 ± 0.01 (1)
A/Missouri/12/2012‡	N1-Classical	0.24 ± 0.06 (1)	7.50 ± 0.73 (17)	0.07 ± 0.01 (1)	0.14 ± 0.02 (1)
A/Arkansas/14/2013	N1-Classical	0.26 ± 0.04 (1)	1.25 ± 0.24 (3)	0.11 ± 0.02 (1)	0.31 ± 0.07 (1)
A/Arkansas/15/2013	N1-Classical	0.29 ± 0.08 (1)	0.90 ± 0.13 (2)	0.12 ± 0.02 (1)	0.41 ± 0.09 (2)
A/Minnesota/33/2014	N1-Classical	0.23 ± 0.00 (1)	1.11 ± 0.22 (3)	0.11 ± 0.02 (1)	0.32 ± 0.01 (1)
A/Ohio/09/2015	N1-Classical	0.27 ± 0.04 (1)	0.50 ± 0.14 (1)	0.12 ± 0.03 (1)	0.39 ± 0.19 (1)
A/Iowa/39/2015	N1-Classical	0.22 ± 0.02 (1)	0.85 ± 0.14 (2)	0.10 ± 0.01 (1)	0.32 ± 0.02 (1)
A/Iowa/33/2017	N1-pdm09	0.13 ± 0.03 (1)	0.10 ± 0.03 (0.2)	0.05 ± 0.01 (1)	0.17 ± 0.05 (1)
A/Michigan/288/2019	N1-pdm09	0.27 ± 0.02 (1)	0.43 ± 0.03 (1)	0.06 ± 0.01 (1)	0.19 ± 0.01 (1)
A/Iowa/22/2020	N1-pdm09	0.17 ± 0.02 (1)	0.30 ± 0.03 (1)	0.07 ± 0.00 (1)	0.20 ± 0.02 (1)
A/Iowa/23/2020§	N1-pdm09	0.18 ± 0.03 (1)	0.27 ± 0.01 (1)	0.07 ± 0.01 (1)	0.22 ± 0.02 (1)
A/Iowa/01/2021	N1-pdm09	0.16 ± 0.02 (1)	0.19 ± 0.01 (0.4)	0.07 ± 0.01 (1)	0.20 ± 0.01 (1)
A/Iowa/02/2021¶	N1-pdm09	0.33 ± 0.02 (2)	1.05 ± 0.18 (2)	0.20 ± 0.01 (3)	0.57 ± 0.03 (3)
A/North Carolina/01/2021	N1-pdm09	0.18 ± 0.01 (1)	0.22 ± 0.02 (1)	0.07 ± 0.01 (1)	0.20 ± 0.03 (1)
A/North Dakota/12226/2021	N1-pdm09	0.18 ± 0.02 (1)	0.22 ± 0.02 (1)	0.07 ± 0.01 (1)	0.20 ± 0.01 (1)
A/Wisconsin/03/2021	N1-Classical	0.22 ± 0.03 (1)	0.48 ± 0.23 (1)	0.10 ± 0.01 (1)	0.37 ± 0.09 (1)
Median IC_{50}, A(H1N2)v (n = 14)†		*0.55*	*0.20*	*0.16*	*0.92*
A/Michigan/09/2007	N2–2002	0.37 ± 0.10 (1)	0.11 ± 0.02 (1)	0.11 ± 0.01 (1)	0.39 ± 0.02 (0.4)
A/Ohio/24/2017	N2–2002	0.25 ± 0.02 (1)	0.10 ± 0.02 (1)	0.10 ± 0.01 (1)	0.39 ± 0.07 (0.4)
A/Ohio/35/2017	N2–2002	0.52 ± 0.11 (1)	0.20 ± 0.02 (1)	0.19 ± 0.01 (1)	0.63 ± 0.09 (1)
A/California/62/2018	N2–1998	0.74 ± 0.07 (1)	0.20 ± 0.03 (1)	0.16 ± 0.04 (1)	1.19 ± 0.08 (1)
A/California/63/2018	N2–1998	0.53 ± 0.07 (1)	0.17 ± 0.02 (1)	0.16 ± 0.02 (1)	1.25 ± 0.22 (1)
A/Michigan/382/2018	N2–1998	0.48 ± 0.05 (1)	0.22 ± 0.01 (1)	0.16 ± 0.01 (1)	0.89 ± 0.11 (1)
A/Michigan/383/2018	N2–1998	0.46 ± 0.07 (1)	0.21 ± 0.06 (1)	0.15 ± 0.04 (1)	0.90 ± 0.16 (1)
A/Michigan/384/2018	N2–1998	0.88 ± 0.01 (2)	0.27 ± 0.01 (1)	0.18 ± 0.01 (1)	1.00 ± 0.00 (1)
A/Ohio/25/2018	N2–1998	0.68 ± 0.05 (1)	0.23 ± 0.03 (1)	0.16 ± 0.01 (1)	0.95 ± 0.09 (1)
A/Iowa/04/2021	N2–1998	0.47 ± 0.01 (1)	0.16 ± 0.02 (1)	0.10 ± 0.01 (1)	0.63 ± 0.04 (1)
A/Ohio/28/2022	N2–1998	0.58 ± 0.10 (1)	0.28 ± 0.09 (1)	0.19 ± 0.03 (1)	1.16 ± 0.20 (1)
A/Michigan/48/2023	N2–1998	0.77 ± 0.06 (1)	0.19 ± 0.01 (1)	0.18 ± 0.01 (1)	1.10 ± 0.01 (1)
A/Montana/28/2023	N2–1998	0.68 ± 0.04 (1)	0.19 ± 0.01 (1)	0.16 ± 0.01 (1)	0.97 ± 0.04 (1)
A/Pensylvania/27/2024	N2–1998	0.57 ± 0.11 (1)	0.31 ± 0.07 (1)	0.12 ± 0.02 (1)	0.54 ± 0.06 (1)
Median IC_{50}, A(H3N2)v (n = 21)†		*0.37*	*0.11*	*0.12*	*0.51*
A/Ohio/83/2012#	N2–2002	0.42 ± 0.12 (1)	0.11 ± 0.02 (1)	0.13 ± 0.02 (1)	0.51 ± 0.05 (1)
A/Ohio/88/2012#	N2–2002	34.78 ± 5.40 (94)	5.09 ± 1.01 (46)	0.20 ± 0.02 (2)	4.45 ± 0.52 (9)
A/Iowa/04/2013	N2–2002	0.50 ± 0.10 (1)	0.17 ± 0.03 (1)	0.20 ± 0.03 (2)	0.73 ± 0.01 (1)
A/Ohio/02/2014	N2–2002	0.54 ± 0.12 (1)	0.18 ± 0.03 (2)	0.19 ± 0.05 (2)	0.64 ± 0.07 (1)
A/Ohio/4319/2014	N2–2002	0.60 ± 0.09 (2)	0.20 ± 0.04 (2)	0.21 ± 0.03 (2)	0.70 ± 0.04 (1)
A/Wisconsin/24/2014	N2–2002	0.59 ± 0.01 (2)	0.20 ± 0.02 (2)	0.20 ± 0.02 (2)	0.75 ± 0.05 (1)
A/Michigan/39/2015	N2–2002	0.37 ± 0.20 (1)	0.10 ± 0.04 (1)	0.12 ± 0.05 (1)	0.58 ± 0.15 (1)
A/New Jersey/53/2015	N2–1998	0.41 ± 0.09 (1)	0.15 ± 0.01 (1)	0.14 ± 0.02 (1)	0.74 ± 0.16 (1)
A/Michigan/83/2016	N2–2002	0.31 ± 0.03 (1)	0.12 ± 0.06 (1)	0.11 ± 0.01 (1)	0.47 ± 0.03 (1)
A/Michigan/84/2016	N2–2002	0.37 ± 0.05 (1)	0.11 ± 0.03 (1)	0.13 ± 0.02 (1)	0.49 ± 0.02 (1)
A/Ohio/27/2016	N2–2002	0.29 ± 0.04 (1)	0.09 ± 0.02 (1)	0.10 ± 0.02 (1)	0.44 ± 0.02 (1)
A/Ohio/28/2016	N2–2002	0.31 ± 0.07 (1)	0.11 ± 0.04 (1)	0.10 ± 0.02 (1)	0.40 ± 0.03 (1)
A/North Dakota/19/2017	N2–2002	0.32 ± 0.05 (1)	0.12 ± 0.02 (1)	0.12 ± 0.04 (1)	0.39 ± 0.07 (1)
A/Ohio/13/2017	N2–2002	0.22 ± 0.02 (1)	0.09 ± 0.00 (1)	0.10 ± 0.00 (1)	0.29 ± 0.01 (1)
A/Ohio/14/2017	N2–2002	0.22 ± 0.01 (1)	0.09 ± 0.00 (1)	0.09 ± 0.00 (1)	0.27 ± 0.01 (1)
A/Ohio/15/2017	N2–2002	0.23 ± 0.02 (1)	0.12 ± 0.01 (1)	0.11 ± 0.00 (1)	0.30 ± 0.09 (1)
A/Ohio/29/2017	N2–2002	0.28 ± 0.05 (1)	0.11 ± 0.00 (1)	0.11 ± 0.02 (1)	0.34 ± 0.07 (1)
A/Hawaii/28/2020	N2–1998	0.56 ± 0.12 (2)	0.10 ± 0.01 (1)	0.17 ± 0.00 (1)	1.46 ± 0.21 (3)
A/Wisconsin/01/2021	N2–2002	0.31 ± 0.09 (1)	0.09 ± 0.01 (1)	0.11 ± 0.02 (1)	0.52 ± 0.08 (1)
A/West Virginia/24/2022	N2–2002	0.29 ± 0.05 (1)	0.10 ± 0.00 (1)	0.12 ± 0.01 (1)	0.40 ± 0.05 (1)
A/West Virginia/25/2022	N2–2002	0.44 ± 0.07 (1)	0.20 ± 0.00 (2)	0.22 ± 0.01 (2)	0.79 ± 0.13 (2)
A/West Virginia/26/2022	N2–2002	0.57 ± 0.04 (2)	0.19 ± 0.01 (2)	0.19 ± 0.01 (2)	0.76 ± 0.01 (1)
Reference seasonal viruses**f					
A/Illinois/45/2019 (H1N1)pdm09	n/a	0.15 ± 0.03	0.19 ± 0.03	0.05 ± 0.01	0.16 ± 0.03
A/Pennsylvania/46/2015 (H3N2)	n/a	0.24 ± 0.04	0.15 ± 0.03	0.09 ± 0.02	0.36 ± 0.06

*Each virus was tested in ≥3 independent experiments to determine the mean IC_{50} value. IC_{50}, 50% inhibitory concentration, n/a, not applicable; NA, neuraminidase.

†Variant viruses lacking NA substitutions of interest were used to determine the median IC_{50} (the baseline susceptibility). The fold-change in IC_{50}s relative to the subtype-specific median IC_{50} is shown.

‡A/Missouri/12/2012 (H1N1)v contains an I427V substitution in its NA segment (EPI395312).

§A/Iowa/23/2020 (H1N1)v virus is the closest wild-type NA sequence-match of A/Iowa/02/2021 (H1N1)v virus.

¶A/Iowa/02/2021 (H1N1)v contains an S247N substitution in its NA gene segment; additionally contains N188S and V398I NA substitutions compared to A/Iowa/23/2020 (H1N1)v.

#These viruses were previously reported and tested in NI assay (*3*).

**Source: CDC Neuraminidase Inhibitor Susceptibility Reference Virus Panel (IRR: FR-1755 ver3).

Appendix Table 3. Subtype-specific baloxavir susceptibility of U.S. variant influenza viruses in cell culture-based assay IRINA*

Variant influenza A virus	PA lineage	28	Amino acid change in PA-CEN domain Other residues	EC_{50}±SD, nM (fold-change)
Median EC_{50}, A(H1N1)v (n = 15)†				***0.75***
A/Texas/14/2008	TRIG	S	-	0.93 ± 0.38 (1)
A/California/07/2009	pdm09	P	E18G, Q57R, T85I, V118I, G186S, R204K	1.32 ± 0.24 (2)
A/Missouri/12/2012	pdm09	P	Q57R, I61V, T85I, V118I, T151N, G186S, R204K	1.13 ± 0.04 (2)
A/Arkansas/14/2013	pdm09	P	I13V, Q57R, I61V, T85I, G99R, V118I, G186S, R204K	0.76 ± 0.12 (1)
A/Arkansas/15/2013	pdm09	P	I13V, Q57R, I61V, T85I, G99R, V118I, G186S, R204K	0.65 ± 0.12 (1)
A/Minnesota/33/2014	TRIG	S	F35L, A70V	0.50 ± 0.12 (1)
A/Ohio/09/2015	TRIG	S	-	0.54 ± 0.14 (1)
A/Iowa/39/2015	pdm09	P	Q57R, I61V, P68S, T85I, V118I, G186S, R204K	0.52 ± 0.13 (1)
A/Iowa/33/2017 clone 1‡	pdm09	P	Q57R, T85I, V118I, G186S, R204K, T208K	1.33 ± 0.07 (2)
A/Iowa/33/2017 clone 2‡	pdm09	P	**I38M,** Q57R, T85I, V118I, G186S, R204K, T208K	19.98 ± 2.17 (27)
A/Michigan/288/2019	LAIV	P	Q57R, V100I, V118I	2.35 ± 0.07 (3)
A/Iowa/22/2020	pdm09	P	Q57R, V63L, T85I, V100I, V118I, G186S	1.36 ± 0.30 (2)
A/Iowa/23/2020	pdm09	P	Q57R, V63L, T85I, V100I, V118I, G186S	0.75 ± 0.07 (1)
A/Iowa/01/2021	pdm09	P	Q57R, V63L, T85I, V100I, V118I, G186S	1.04 ± 0.31 (1)
A/Iowa/02/2021	pdm09	P	Q57R, I61V, T85I, N115D, V118I, I129V, G186S	0.75 ± 0.16 (1)
A/North Carolina/01/2021	pdm09	P	Q57R, V63L, T85I, V100I, V118I, G186S, R204K	0.69 ± 0.09 (1)
A/North Dakota/12226/2021	TRIG	S	G66S, T97A, R204K	1.58 ± 0.23 (2)
A/Wisconsin/03/2021	pdm09	P	F4L, Q57R, I61V, V118I, G186S, R204K	0.44 ± 0.12 (1)
Median EC_{50}, A(H1N2)v (n = 14)†				***0.98***
A/Michigan/09/2007	TRIG	S	-	1.55 ± 0.13 (2)
A/Ohio/24/2017	pdm09	P	Q57R, T85I, G186S, R204K	0.87 ± 0.11 (1)
A/Ohio/35/2017	TRIG	P	E59G, M86V, V90I	1.03 ± 0.16 (1)
A/California/62/2018	TRIG	S	V44I, Q193L	2.32 ± 0.16 (2)
A/California/63/2018	TRIG	S	V44I, E59D	1.90 ± 0.46 (2)
A/Michigan/382/2018	TRIG	P	V44I, D55N, G66D, S184G	0.64 ± 0.18 (1)
A/Michigan/383/2018	TRIG	P	V44I, D55N, G66D, S184G	0.45 ± 0.12 (1)
A/Michigan/384/2018	TRIG	P	V44I, D55N, G66D, S184G	1.02 ± 0.11 (1)
A/Ohio/25/2018	TRIG	P	V44I, D55N, G66D, V100I, S184G	0.98 ± 0.05 (1)
A/Iowa/04/2021	TRIG	P	V44I, D55N, G66D	0.74 ± 0.05 (1)
A/Ohio/28/2022	TRIG	P	V44I, D55N, G66D, T85A	0.97 ± 0.18 (1)
A/Michigan/48/2023	TRIG	P	V44I, D55N, G66D, T85A	0.70 ± 0.09 (1)
A/Montana/28/2023	TRIG	P	V44I, D55N, G66D, T85A	0.94 ± 0.08 (1)
A/Pennsylvania/27/2024	TRIG	S	T85A, K142R, R204K	1.32 ± 0.22 (1)
Median EC_{50}, A(H3N2)v (n = 21)†				***1.27***
A/Ohio/83/2012	TRIG	S	-	1.54 ± 0.23 (1)
A/Ohio/88/2012	TRIG	S	-	1.03 ± 0.25 (1)
A/Iowa/04/2013	pdm09	P	Q57R, I61V, T85I, V118I, I120V, I129L, G186S, R204K	1.38 ± 0.38 (1)
A/Ohio/02/2014	TRIG	S	V100I	1.53 ± 0.16 (1)
A/Ohio/4319/2014	TRIG	S	V100I	1.46 ± 0.12 (1)
A/Wisconsin/24/2014	TRIG	S	M86I	1.23 ± 0.19 (1)
A/Michigan/39/2015	TRIG	S	V90I	0.80 ± 0.12 (1)
A/New Jersey/53/2015§	TRIG	S	**E199G**	1.50 ± 0.17 (1)
A/Michigan/83/2016	TRIG	S	I13V, T151N, T208S	1.69 ± 0.46 (1)
A/Michigan/84/2016	TRIG	S	-	1.53 ± 0.31 (1)
A/Ohio/27/2016	TRIG	S	I13V, T151N, T208S	0.97 ± 0.08 (1)
A/Ohio/28/2016	TRIG	S	I13V, T151N, R192L, T208S	0.51 ± 0.10 (1)
A/North Dakota/19/2017	TRIG	S	-	1.00 ± 0.29 (1)
A/Ohio/13/2017	TRIG	S	-	1.09 ± 0.18 (1)
A/Ohio/14/2017	TRIG	S	-	1.85 ± 0.33 (2)
A/Ohio/15/2017	TRIG	S	-	1.78 ± 0.20 (2)
A/Ohio/29/2017	TRIG	S	-	1.27 ± 0.21 (1)
A/Hawaii/28/2020	LAIV	P	D3A, Q57R, V118I	1.15 ± 0.07 (1)
A/Wisconsin/01/2021	TRIG	S	T97A, V100A, I201V, T208S	0.42 ± 0.02 (1)
A/West Virginia/24/2022	TRIG	S	V14I, V100I	1.35 ± 0.34 (1)
A/West Virginia/25/2022	TRIG	S	V14I, V100I	1.47 ± 0.47 (1)
A/West Virginia/26/2022	TRIG	S	V14I, V100I	0.92 ± 0.02 (1)
***Reference seasonal* viruses¶**				
A/Illinois/08/2018 (H1N1)pdm09	n/a	n/a	n/a	1.24 ± 0.17
A/Louisiana/50/2017 (H3N2)	n/a	n/a	n/a	1.08 ± 0.17

*PA-CEN domain encompasses the N terminal 1 - 209 aa residues in PA protein (*4*). Sequences were compared to that of A/Texas/14/2008 (H1N1)v containing PA gene segment from TRIG-lineage and serine (S) at residue 28. Each virus was tested in ≥3 independent experiments to determine the mean EC_{50} value (50% effective concentration, nM); SD, standard deviation. Dash lines indicate similar amino acid sequence with that of A/Texas/14/2008 (H1N1)v. Amino acid changes in bold indicate known or suspected molecular markers that affect baloxavir susceptibility. aEC_{50}, 50% effective concentration; IRINA, influenza replication inhibition neuraminidase-based assay; n/a, not applicable; PA, polymerase acidic; PA-CEN, PA cap-dependent endonuclease; TRIG, triple-reassortment internal gene.

†Variant viruses lacking PA substitutions of interest were used to determine the subtype-specific median EC_{50} (baseline susceptibility). The fold change in EC_{50}s relative to the median EC_{50} is shown.

‡These viruses were previously tested with baloxavir (*1*).

§A/New Jersey/53/2015 (H3N2)v contains an E199G PA substitution.

¶Source: CDC Baloxavir Susceptibility Reference Virus Panel (IRR: FR-1678 ver1.1).

Appendix Table 4. Sequence variations in the mAb epitopes mapped at the N terminal and C-terminal portion of HA1 and N -terminal portion of HA2 of tested variant viruses*

		Amino acid variations in antibody epitope																
		HA1 (H3 numbering)							HA2									
**Influenza A virus**	**HA clade**	**Gly‡**	**39**	**40**	**42**	**291**	**292**	**320**	**18**	**38**	**39**	**43**	**45**	**46**	**48**	**49**	**52**	**HA acc. ID†**
A(H1N1)v (n = 7)																		
A/California/07/2009	1A.3.3.2	No	S	V	L	S	L	L	V	L	K	N	I	D	I	T	V	EPI1161425
A/North Carolina/01/2021	1A.3.3.2	No	-	-	-	-	-	-	-	-	-	-	-	-	-	-	-	EPI1869574
A/North Dakota/12226/2021	1A.3.3.3-c1	No	-	-	-	-	-	-	I	-	-	-	-	-	-	-	-	EPI1918841
A/Missouri/12/2012	1A.3.3.3-c3	No	-	-	-	-	-	-	I	-	-	-	-	-	-	-	-	EPI395313
A/Arkansas/14/2013	1A.3.3.3-c3	No	-	-	-	-	-	-	I	-	-	-	-	-	-	-	-	EPI471102
A/Arkansas/15/2013	1A.3.3.3-c3	No	-	-	-	-	-	-	I	-	-	-	-	-	-	-	-	EPI482785
A/Wisconsin/03/2021	1A.3.3.3-c3	No	-	-	-	-	-	-	I	-	-	S	-	-	-	-	-	EPI1868840
A(H1N2)v (n = 7)																		
A/Ohio/24/2017	1A.1.1.3	No	-	-	-	-	-	-	I	Q	-	R	-	-	-	-	-	EPI1056725
A/Pennsylvania/27/2024	1A.1.1.3	No	-	-	-	-	-	-	-	Q	-	R	-	-	-	-	-	EPI3171496
A/Michigan/382/2018	1B.2.1	No	-	-	-	-	-	-	-	Q	Q	I	-	N	-	-	-	EPI1271034
A/Ohio/35/2017	1B.2.1	No	-	-	-	D	-	-	-	Q	-	-	-	N	-	-	-	EPI1056733
A/Iowa/04/2021	1B.2.1	No	-	-	-	N	-	-	-	Q	Q	I	-	N	-	-	-	EPI3215541
A/Ohio/28/2022	1B.2.1	No	-	-	-	-	-	-	-	Q	Q	I	-	N	-	-	-	EPI2193021
A/Michigan/48/2023	1B.2.1	No	-	-	-	-	-	-	-	Q	Q	-	-	N	-	-	-	EPI2687008
A(H3N2)v (n = 8)																		
A/Hawaii/28/2020	1990.1	Yes	A	T	M	D	K	M	-	-	-	A	-	-	V	N	L	EPI1804523
A/Iowa/04/2013	1990.4.a	Yes	A	T	L	D	K	M	-	-	-	A	-	N	-	-	L	EPI516844
A/Wisconsin/24/2014	1990.4.a	Yes	A	T	L	D	K	M	-	-	-	A	-	N	-	-	L	EPI557542
A/Michigan/39/2015	1990.4.a	Yes	A	T	L	D	K	M	-	-	-	A	-	N	-	-	L	EPI642513
A/Ohio/02/2014	1990.4.b1	Yes	A	T	L	D	K	M	-	-	-	A	-	N	-	-	L	EPI539159
A/Ohio/27/2016	2010.1	Yes	A	T	L	D	K	M	-	-	-	A	-	-	-	N	L	EPI881739
A/Ohio/13/2017	2010.1	Yes	A	T	L	D	K	M	-	-	-	A	-	-	-	N	L	EPI1056653
A/Wisconsin/01/2021	2010.1	Yes	A	T	L	D	K	M	-	-	-	T	-	-	-	N	L	EPI1843130
Seasonal reference viruses§																		
A/Illinois/08/2018 (H1N1)pdm09	n/a	No	-	-	D	-	-	-	-	-	-	-	-	-	-	-	-	EPI1259741
A/Louisiana/50/2017 (H3N2)	n/a	Yes	A	T	L	D	K	M	-	-	-	A	-	-	-	N	L	EPI1259757

*Epitopes are based on those mapped by Corti et al. (*5*) and Dreyfuss et al. (*6*). Shown are residues with sequence variations only. All other epitopes were conserved for all viruses examined. Dashes indicate similar amino acid sequence with that of A/California/07/2009 (H1N1)pdm09.Yellow highlighting indicates FI6v3-specific epitopes; green highlighting indicates CR9114-specific epitopes; blue highlighting indicates common epitopes for both mAbs. HA, hemagluttinin; mAb, monoclonal antibody; n/a, not applicable.
†HA gene sequences deposited in the GISAID database (accessed in April 2024). Sequences were aligned and analyzed using MAFFT ver7 program. Dash lines indicate similar sequence as reference.
‡Presence (Yes) or absence (No) of a potential glycosylation (Gly) site at HA1 position 38.
§Source: CDC antiviral susceptibility reference virus panels (FR-1678 ver1.1).

Appendix Table 5. Alignment of the H1 HA of clade 1B.2.1 (delta 2) A(H1N2)v viruses tested for antigenic relatedness by HI and IRINA*

A(H1N2)v viruses	HA1 (H1 numbering)																																							
	**5**	**16**	**36**	**69**	**71**	**72**	**73**	**82**	**83**	**89**	**96**	**102**	**104**	**113**	**119**	**120**	**121**	**130**	**156**	**166**	**168**	**169**	**170**	**173**	**185**	**189**	**193**	**199**	**205**	**208**	**209**	**215**	**216**	**224**	**237**	**256**	**260**	**276**	**277**	**289**	**310**
Reference CVV†																																									
A/Ohio/35/2017	V	V	T	L	I	P	K	T	P	T‡	T	R	Q	R	K	D	G	S	G	T	K	K	E	V	I	K	H	V	H	K	K	A	R	E	G	E	G	N	A	D	T
Test viruses																																									
A/Michigan/383/2018	-	-	-	-	N	S	-	-	S	-	E	-	-	K	-	K	S	T	N	-	-	E	G	-	M	R	-	-	-	R	R	-	K	-	-	-	S	-	T	-	A
A/Iowa/04/2021	-	-	N	-	T	S	Q	-	S	-	E	-	H	K	-	K	S	T	N	I	-	E	G	-	M	-	-	-	-	R	R	-	K	-	-	K	S	-	T	N	A
A/Ohio/28/2022	-	-	R	-	T	S	-	-	S	-	E	K	-	-	-	K	S	T	N	-	-	-	G	-	M	R	-	I	Y	R	R	V	K	G	-	-	S	D	T	-	A
A/Montana/28/2023	I	I	-	M	-	S	-	K	-	A	E	-	-	-	E	K	S	T	N	-	T	-	-	I	-	R	N	-	-	R	R	-	K	G	K	-	-	-	-	-	-
A/Michigan/48/2023	I	I	-	M	-	S	-	K	-	A	E	-	-	-	E	K	S	T	N	-	T	-	-	I	-	R	N	-	-	R	R	-	K	G	K	-	-	-	-	-	-
Reference seasonal A(H1N1)pdm09 CVV§																																									
A/Victoria/2570/2019	I	-	K	S	S	T	A	T	S	-	I	R	-	-	-	T	S	N	K	I	D	-	G	-	-	Q	Q	-	R	-	-	-	T	-	-	T	D	-	T	-	-

*HA gene sequences deposited in the GISAID database (accessed on April 2024). Sequences were aligned and analyzed using MAFFT ver7 program. Blue highlighting indicates Cb antigenic sites; yellow highlighting indicates Ca antigenic site; green highlighting indicates Sb antigenic site. Gray highlighting indicates amino acid differences specific for the 2023 A(H1N2)v viruses. Dashes indicate similar sequence as reference (CVV for clade 1B.2.1). CVV, candidate vaccine virus; HA, hemagglutinin; HI, hemagglutination inhibition; IRINA, influenza replication inhibition neuraminidase-based assay.
†Recommended A(H1N2)v candidate vaccine virus for clade 1B.2.1 (delta 2) in 2018 (*7*).
‡Underlined residue indicate the presence of a potential glycosylation motif (residue 87–89).
§Recommended seasonal A(H1N1)pdm09 candidate vaccine virus from 2021–present (*8*).

References

1. Mishin VP, Patel MC, Chesnokov A, De La Cruz J, Nguyen HT, Lollis L, et al. Susceptibility of influenza A, B, C, and D viruses to baloxavir. Emerg Infect Dis. 2019;25:1969–72. PubMed https://doi.org/10.3201/eid2510.190607

2. Sleeman K, Mishin VP, Guo Z, Garten RJ, Balish A, Fry AM, et al. Antiviral susceptibility of variant influenza A(H3N2)v viruses isolated in the United States from 2011 to 2013. Antimicrob Agents Chemother. 2014;58:2045–51. PubMed https://doi.org/10.1128/AAC.02556-13

4. Dias A, Bouvier D, Crépin T, McCarthy AA, Hart DJ, Baudin F, et al. The cap-snatching endonuclease of influenza virus polymerase resides in the PA subunit. Nature. 2009;458:914–8. PubMed https://doi.org/10.1038/nature07745

5. Corti D, Voss J, Gamblin SJ, Codoni G, Macagno A, Jarrossay D, et al. A neutralizing antibody selected from plasma cells that binds to group 1 and group 2 influenza A hemagglutinins. Science. 2011;333:850–6. PubMed https://doi.org/10.1126/science.1205669

6. Dreyfus C, Laursen NS, Kwaks T, Zuijdgeest D, Khayat R, Ekiert DC, et al. Highly conserved protective epitopes on influenza B viruses. Science. 2012;337:1343–8. PubMed https://doi.org/10.1126/science.1222908

7. World Health Organization. Antigenic and genetic characteristics of zoonotic influenza viruses and development of candidate vaccine viruses for pandemic preparedness [cited 2024 Jun 17]. https://www.who.int/docs/default-source/influenza/who-influenza-recommendations/vcm-northern-hemisphere-recommendation-2018-2019/201802-zoonotic-vaccinevirusupdate.pdf

8. World Health Organization. Recommended composition of influenza virus vaccines for use in the 2021- 2022 northern hemisphere influenza season [cited 2024 Jun 17]. https://cdn.who.int/media/docs/default-source/influenza/who-influenza-recommendations/vcm-northern-hemisphere-recommendation-2021-2022/202102_recommendation.pdf
